# Immediate post-injury HMGB1 neutralization prevents synaptic dysfunction in burn and hindlimb unloaded rats

**DOI:** 10.3389/fimmu.2026.1867953

**Published:** 2026-06-23

**Authors:** Sravan Gopalkrishna Shetty Sreenivasa Murthy, Gábor Törő, Allison Wyrick, Amina El Ayadi, Steven E. Wolf, Nisha J. Garg, Balaji Krishnan, Juquan Song

**Affiliations:** 1 Mitchell Center for Neurodegenerative Disease, The University of Texas Medical Branch at Galveston, TX, United States; 2 Department of Neurology, The University of Texas Medical Branch at Galveston, TX, United States; 3 Department of Surgery, The University of Texas Medical Branch at Galveston, TX, United States; 4 Department of Microbiology & Immunology, The University of Texas Medical Branch at Galveston, TX, United States

**Keywords:** electrophysiology, hippocampus, immobilization, long-term potentiation, thermal injury

## Abstract

**Introduction:**

Severe burns are known to provoke neuroinflammation and contribute to cognitive deficits, but the mechanism of action remains poorly understood. We hypothesize that early release of extracellular high mobility group box 1 (HMGB1), a key driver for inflammation, triggers the burn−induced hyperinflammation and associated synaptic dysfunction. Unavoidable immobilization prolongs inflammation and worsens burn outcomes. We examined the therapeutic potential of an anti−HMGB1 neutralizing antibody in improving the neurological outcomes in burned and immobilized rats.

**Methods:**

Adult male rats received >30% total body surface area (TBSA) scald burns. After injury, animals received either no treatment, chicken anti−HMGB1, or isotype control IgY antibody (2 mg/kg, intraperitoneal or combined with subcutaneous Alzet pump delivery). Burn−injured, treated rats underwent 14 days of hindlimb unloading (HLU) in metabolic cages, followed by 7 days of mobile recovery. Burned rats without treatment were pair−fed and housed in standard cages without suspension, as were sham−burn controls. All animals were euthanized 21 days post−injury for sample collection. Hippocampal synaptic integrity was evaluated using field electrophysiological recordings at Schaffer collateral synapses.

**Results:**

Burn wound size was significantly increased in rats subjected to hindlimb unloading, accompanied by elevated IL−10 and IL−1β levels. These alterations were mitigated by anti−HMGB1 antibody treatment. Furthermore, anti−HMGB1 administration moderated the activated CD4^+^T cells and NK cells response. Electrophysiological analysis of hippocampal slices showed that burn−HLU rats treated with vehicle, exhibited pronounced postsynaptic hyperexcitability and left−shifted presynaptic excitability curves, consistent with synaptic dysfunction. These animals also demonstrated disrupted slope profiles and loss of basal-excitatory resolution, indicating heightened excitability and increased vulnerability to degeneration. Anti−HMGB1 antibody treatment restored normal potentiation patterns and preserved both pre− and postsynaptic function in burned rats with 14-days hindlimb unloading.

**Discussion:**

Prolonged immobilization provoked pre− and post-synaptic hyperexcitability at Schaffer collateral synapses and exacerbated burn−induced brain impairment in rats. Early reduction of systemic HMGB1 activity protected against inflammation, preserved the hippocampal synaptic plasticity, and restored long−term potentiation and hippocampal integrity following burn injury and hindlimb unloading in rats.

## Graphical Abstract

**Figure:**
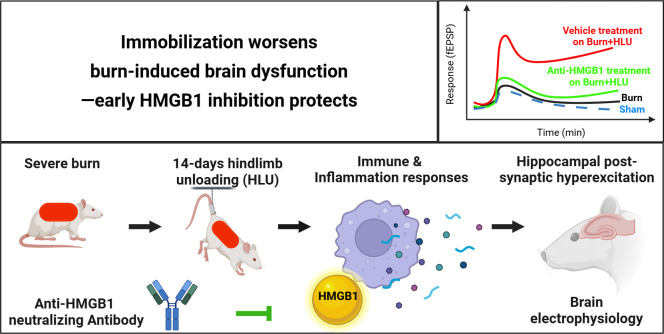

## Highlights

- Severe burns combined with prolonged immobilization induce hyperinflammation and hippocampal synaptic dysfunction, manifested as pre− and post−synaptic hyperexcitability and impaired long−term potentiation.
- HMGB1−driven inflammation is exacerbated by hindlimb unloading, leading to increased burn wound size, elevated inflammatory cytokines (IL−1β, IL−10), and heightened immune activation, worsening both peripheral and central outcomes.
- Anti−HMGB1 neutralizing antibody treatment mitigates systemic inflammation and preserves hippocampal function.

## Background

Thermal burns are the 4^th^ most common type of trauma as a global health concern, causing 6.6–12 million medical encounters and up to 300,000 deaths annually (1) (2). The Centers for Disease Control (CDC) and American Burn Association also document significant burn incidences in the USA, with estimated 500,000 burn records and ~3,800 deaths per year (3). The impact of burns extends far beyond acute injury and immediate mortality. For example, patients sustaining ≥30% total body surface area (TBSA) burns experience profound metabolic disruption, losing up to 25% of total body mass in the acute phase (4, 5), and continue to suffer significant, long−lasting impairments for years after the initial insult (6). Severe burn injuries cause both physical and psychological changes and lead to devastating outcomes (7). Burn survivors have higher rates of mental−health–related emergency care than the general population (8). Multiple studies have demonstrated that burn injury increased the lifelong vulnerability to post−traumatic stress disorder (PTSD), anxiety, depression, suicidal thoughts, and substance use disorders (9). Post−burn psychological changes and mental recovery are critical for improving patients’ quality of life. The underlying mechanisms and effective treatments for these mental health concerns, however, remain to be addressed.

Severe burn injury initiates a cascade of pathological processes that can profoundly compromise the integrity of the central nervous system (CNS). Burn injury signals systemic inflammatory response (10) that disrupts the blood–brain barrier (11) and facilitates infiltration of cytokines and immune cells into the CNS (12). These inflammatory mediators may disturb the oligodendrocyte–neuron interactions (13) and promote demyelination (14), resulting in persistent structural and functional neurological impairment. In parallel, synaptic plasticity—encompassing activity−dependent synaptic potentiation and structural remodeling—plays a central role in maintaining hippocampal learning, memory, and adaptive cognitive function (15) (16)., When these mechanisms are compromised, as observed in traumatic brain injury (17), Alzheimer’s disease (18), and aging (19), the synaptic efficacy, dendritic spine density, and network connectivity are reduced. Collectively, these alterations contribute to measurable and progressive cognitive deficits.

The transition from acute burn to chronic illness begins with profound systemic disruption initiated by the injury. Local tissue destruction rapidly escalates into widespread inflammation, driving multi−system stress responses (20). These disturbances often persist long after wound closure, evolving into chronic systemic dysregulation that can last for decades. Emerging evidence points to pathogen− and damage−associated molecular patterns (PAMPs and DAMPs) as key drivers of this prolonged state, in part by activating persistent CNS−mediated stress pathways that influence multiple organ systems (21). Among DAMPs, HMGB1 has gained particular attention. When released extracellularly, HMGB1 functions as a potent inflammatory signal and biomarker, interacting with the immune system or directly altering cellular homeostasis through the RAGE–TLR4 signaling axis (22). HMGB1 is increasingly recognized as a promising therapeutic target across a range of diseases, including neurodegenerative diseases (23). Prior work from our group demonstrated that treatment with anti-HMGB1 antibody improved wound healing and muscle preservation following burn injury in rodents (24) (25)., Thus, we propose that early inhibition of HMGB1 activity may prevent burn−induced neuroinflammation and enhance functional recovery.

Clinical context is important to consider when translating our findings, as HMGB1−targeted interventions may improve patient outcomes post-burn. In acute burn care, clinical nursing plans prioritize the ABCDEs (Airway, Breathing, Circulation, Disability, Exposure), along with fluid resuscitation, infection control, and pain management. Any therapy that supports the major etiological processes of wound repair and healing would therefore be considered within the broader framework of comprehensive burn management. Prolonged immobilization is an unavoidable component of early clinical management for severe burn patients, who often require strict physical restriction during the initial 72 hours post-burn, and typically remain hospitalized for extended periods. Longer hospital stays are consistently associated with poorer outcomes (26) (27), Extended bed rest—whether due to clinical necessity, limb suspension, casting, or other reasons—has been shown to suppress protein synthesis to accelerate disuse muscle atrophy (28), and contribute to neurocognitive disturbances (29). Using our established, preclinical burn-injury model with and without 14 days immobilization, we evaluated the potential of anti−HMGB1 neutralizing antibody treatment in preserving the neural synaptic plasticity and mitigating the burn−related inflammation and neurological decline.

## Methods

### Animal protocol: ethical considerations

Sprague Dawley, male rats (251-275g, age: 9–10 weeks, cat# 400SASSD) were purchased from Charles River Laboratories (Wilmington, MA). Animals were housed in groups of three per cage in a temperature- and humidity-controlled facility (approximately 71°F/40% relative humidity) under a 12-hour light/dark cycle (lights on from 06:00 to 18:00). Rats were provided with ad libitum access to standard laboratory chow and drinking water. All experiments were conducted under research protocol (APN# 2404031) approved by the Institutional Animal Care and Use Committee (IACUC). Furthermore, all animal procedures adhered to the guidelines set forth by the National Institutes of Health (NIH) and the Animal Research: Reporting of *In Vivo* Experiments (ARRIVE) standards.

### Animal experiment design and procedures

**Figure 1:**
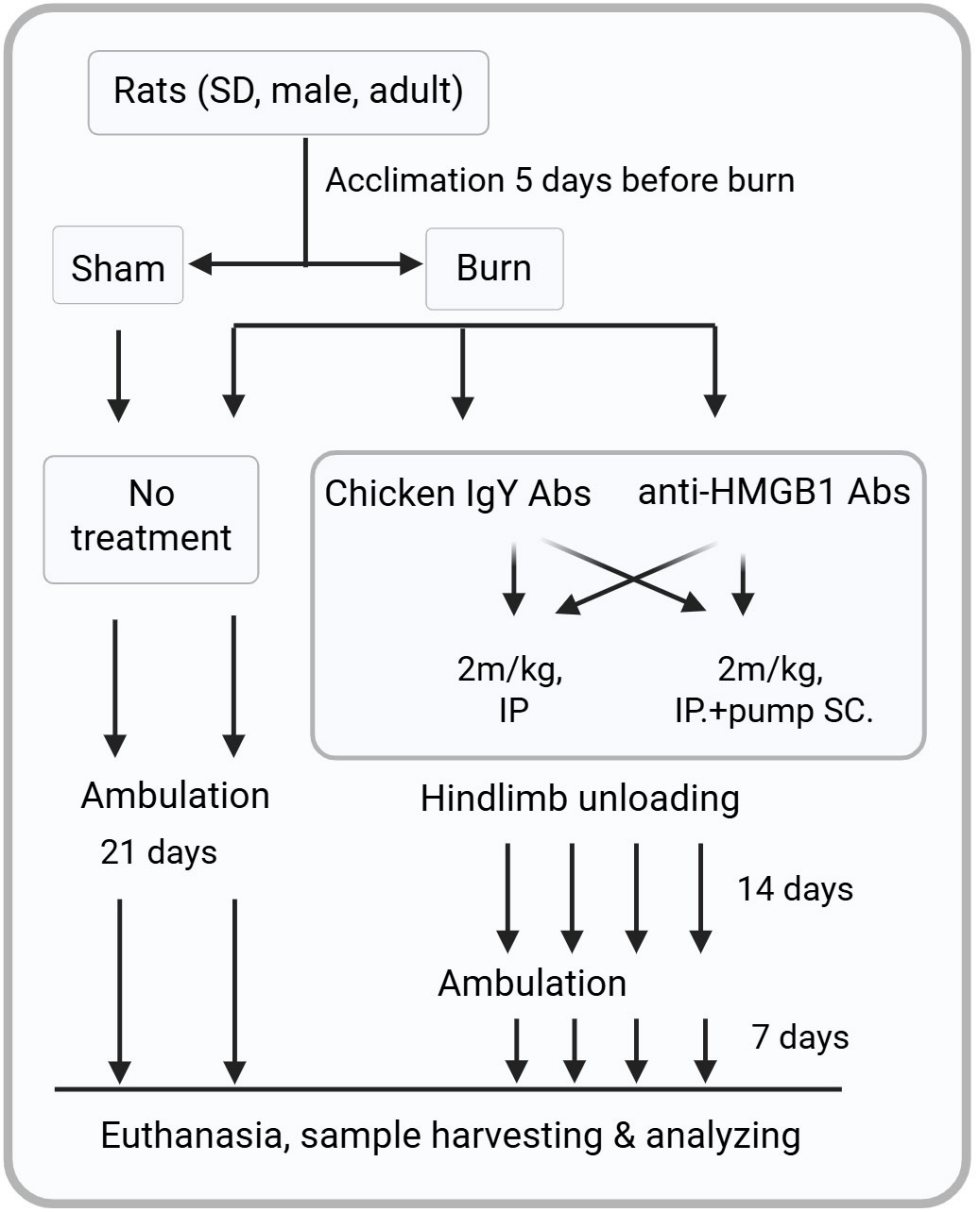
Experiment flow chart. The study included 47 rats. All animals were acclimated in metabolic cages 5 days before procedure and randomly grouped: Sham (n=10) and Burn (n=12) without treatment and kept ambulatory for 21 days. Burn and hindlimb unloaded rats (BH) were administered chicken IgY (control) antibody by IP injection (BHV, n=10) or by IP and subcutaneously (SC) inserted pump (BHVP, n=3). BH groups were treated with IP delivery of anti-HMGB1 antibody (BHH, n=6) or with IP and SC inserted pump (BHHP, n=6). All treated rats were released from tail suspension system at 14 days and allowed to be ambulatory for additional 7 days to meet the end point.

Five days prior to the experiment, rats were individually housed and acclimated to metabolic cages within a controlled housing environment. This environment featured a reversed 12-hour light/dark cycle (lights off from 06:00 to 18:00, lights on from 18:00 to 06:00), with temperature and relative humidity maintained at 78°F and 40%, respectively. Rats were housed on mesh flooring, allowing for unrestricted movement within a cage space of 7.6 x 11 x 12 inches. Ad libitum access to water and standard rodent chow was provided throughout the experimental period. Food consumption and excreta were monitored, and cage bottoms were cleaned daily during the metabolic cage housing period to assess animal condition and response. On the day of the experiment, animals were randomly assigned to experimental groups. Schematics of experimental design are presented in **Figure 1**.

#### Burn procedure

Briefly, rats were anesthetized with 2-4% isoflurane in oxygen for 30 minutes following subcutaneous administration of extended-release buprenorphine (Ethiqa XR, 1.3 mg/kg). After hair removal from the dorsal and ventral trunk surfaces using clippers, animals received a third-degree scald burn covering up to 40% of their total body surface area. Anesthetized rats were positioned on a custom-made mold to expose the designated burn areas. The mold was immersed in 96–100 °C water, exposing the dorsal skin for 10 seconds and the ventral skin for 2 seconds. Each animal received 15 mL of warmed lactated Ringer’s solution intraperitoneally immediately following the burn procedure (25). The surface wound area of burned animals was measured for severity assessment, as described (24). Sham-treated animals underwent an identical procedure, excluding hot water exposure and fluid resuscitation.

#### Treatment procedure

All burned animals received either immediate treatment or no treatment, administered 5 minutes post-burn. The treatment consisted of either a vehicle (2 mg/kg chicken IgY antibody, cat # ST326058471, Tecan, Switzerland) or 2 mg/kg of an anti-HMGB1 chicken IgY neutralizing polyclonal antibody (cat # ST326052233, Tecan). Two distinct treatment delivery routes were employed: 1) Intraperitoneal (IP): A single bolus injection administered intraperitoneally; 2) Combined IP and Subcutaneous Implantation: Three-quarters of the dose was administered via IP injection, with the remaining one-quarter delivered through a slow-release mini-osmotic pump (Alzet #2001, DURECT, Cupertino, CA) implanted subcutaneously at a rate of 1 µL/hr for 7 days. For subcutaneous osmotic pump implantation, mini pumps were pre-filled with 0.15 mL of the antibody solution under sterile conditions. Following the burn procedure, anesthetized rats were maintained in a prone position with continuous 2-4% isoflurane inhalation. The dorsal skin between the scapulae was aseptically prepared using Betadine solution and alcohol swabs. A 0.5 cm incision was made with a scalpel, and a sterile hemostat was used to create a subcutaneous pocket for pump insertion. The pump was then implanted, ensuring it was beneath intact skin but near the burn wound edge. The incision was subsequently closed with skin glue.

#### Hindlimb unloading

Following treatment, rat’s tail was meticulously cleaned with alcohol swabs and benzoin tincture. Tail was then secured with 3M micropore surgical tape before being connected to a harness system, which was a minor modification of a design previously described (30). Isoflurane inhalation was discontinued, and animals were allowed to recover until they were fully conscious and mobile before being returned to their housing cages. The tail-traction system was carefully adjusted to maintain their hindlimbs suspended at approximately 30° head-down tilt. This setup ensured that rats had unimpeded access to food and water, with their hindlimbs remaining clear off the cage floor throughout the experiment. Signs of pain and stress were closely monitored, and buprenorphine (0.01–0.05 mg/kg, subcutaneous) was administered as needed for analgesia. Rats were released from the suspension system on day 14 and allowed full ambulation within their cages for an additional 7 days. Concurrently, rats in the sham and burn/no treatment groups were housed in metabolic cages within the same facility for 21 days until the study endpoint.

#### Tissue collection

At the conclusion of the study, animals were humanely euthanized under deep anesthesia via inhalation of 5% isoflurane overdose. After euthanasia, body weight and burn wound dimensions were recorded. Blood samples were collected via cardiac puncture into BD Vacutainer K3 EDTA tubes (cat # 02-685-2B, ThermoFisher Scientific, Waltham, MA). For peripheral blood mononuclear cell (PBMC) isolation, the EDTA-treated blood samples were carefully layered over an equal volume of histopaque-1077 (Sigma-Aldrich, St. Louis, MO) and centrifuged at 400×g for 30 minutes at room temperature. PBMCs were then harvested from the buffy coat interface for subsequent flow cytometry analysis. Concurrently, blood plasma was isolated and stored at -20 °C. A 200 µL aliquot of plasma was sent to IDEXX BioAnalytics Laboratory (Columbia, Missouri) for quantification of circulatory biomarkers of inflammation with Milliplex Rat Cytokine/Chemokine 10-Plex Panel (RECYMAG65K27-PMX). Immediately following exsanguination, the rat heads were collected. The intact brains were carefully dissected, maintained in artificial cerebrospinal fluid (aCSF) on ice, and promptly used for electrophysiology studies.

### Flow cytometry

Freshly isolated PBMCs were washed with PBS and processed for flow cytometry. The antibodies/markers and gating strategies for phenotypic and functional characterization of innate immune cells and lymphocytes are shown in **Supplementary Table 1**, **Supplementary Figures 1**, **2**. Briefly, PBMCs were incubated for 15 minutes with Fc Block (anti-CD16/CD32) in brilliant-stain buffer (BD Biosciences, San Jose, CA), and washed with staining buffer (00-4222-26, eBioscience). Cells (5x10^4^ per 50μL) were incubated in dark for 30 min at 4 °C with fluorophore-conjugated antibodies to surface molecules. In some experiments, cells were incubated with fixation-permeabilization solution (BD Biosciences) for 20 min, washed, and stained with fluorophore-conjugated antibodies against intracellular molecules for 30 min. Cells were washed and resuspended in staining buffer for flow cytometry analysis.

All samples were visualized and acquired on a LSRII Fortessa Cell Analyzer (BD Biosciences). Unstained cells, cells incubated with isotype matched IgGs (eBiosciene), live/dead stain, and FMO (fluorescence minus one) were included as controls. Data was analyzed using FlowJo software (v.10.9.0, Becton-Dickinson). Briefly, an average of 1×10^5^ of CD45^+^ or CD3+ live cells from all categorical treatments were selected and combined dataset was subjected to generate common t-distributed stochastic neighbor embedding (t-SNE) plots using parameters: iteration=1,000, perplexity=30, learning rate (eta)=1400, KNN algorithm=Exact, and gradient algorithm=Barnes-Hut, which allows visualization of high-dimensional datasets. Cumulative t-SNE maps were applied to individual files to identify treatment-specific cell subsets.

### Electrophysiology studies

Our standard protocol was used as previously described (31) ^(^32^),^ and modified as follows for the rat experiments. During tissue collection, rat brain was removed and placed in chilled, carbogenated (95% O_2_ and 5% CO_2_ gas mixture) NMDG-artificial cerebrospinal (aCSF) fluid (composition in mM: 93 N-Methyl-D-Gluconate, 2.5 KCl, 1.2 NaH_2_PO_4_, 30 NaHCO_3_, 20 C_8_H_18_N_2_O_4_S, 25 C_6_H_12_O_6_, 5 C_6_H_7_O_6_Na, 2 CH_4_N_2_S, 3 C_3_H_3_NaO_3_, 10 MgSO_4_.7H_2_O, 0.5 CaCl_2_.2H_2_O, 12 C_5_H_9_NO_3_S, pH 7.4). Transverse brain sections (400 μm) were made using Compresstome VF-300 (Precisionary Instruments, Greenville, NC). Brain slices were allowed to recover for 10 min in carbogenated NMDG-aCSF at 33 °C. Slices were then maintained at room temperature in a modified carbogenated HEPES-aCSF solution (composition in mM: 92 NaCl, 2.5 KCl, 1.2 NaH_2_PO_4_, 30 NaHCO_3_, 20 C_8_H_18_N_2_O_4_S, 25 C_6_H_12_O_6_, 5 C_6_H_7_O_6_Na, 2 CH_4_N_2_S, 3 C_3_H_3_NaO_3_, 2 MgSO_4_.7H_2_O, 2 CaCl_2_.2H_2_O, 12 C_5_H_9_NO_3_S, pH 7.4). Slices were recorded in carbogenated normal aCSF (naCSF composition in mM: 124 NaCl, 2.5 KCl, 1.2 NaH_2_PO_4_, 24 NaHCO_3_, 5 C_8_H_18_N_2_O_4_S, 13 C_6_H_12_O_6_, 2 MgSO_4_.7H_2_O, 2 CaCl_2_.2H_2_O, pH 7.4). Evoked field excitatory post-synaptic potential (fEPSP) recordings were performed by stimulating the Schaffer collateral pathway (located in stratum radiatum) using a stimulating electrode of ~22 kΩ resistance placed in the CA3 region and glass recording electrodes in the CA1 region. Current stimulation was delivered through a digital stimulus isolation amplifier (A.M.P.I, ISRAEL) and set to elicit a fEPSP approximately 30% of maximum for synaptic potentiation experiments using platinum-iridium tipped concentric bipolar stimulating electrodes (FHC Inc., Bowdoin, ME). The use of platinum iridium wire and diphasic pulses can help minimize electrode polarization (33). Using a horizontal P-97 Flaming/Brown Micropipette puller (Sutter Instruments, Novato, CA), borosilicate glass capillaries were used to pull recording electrodes and filled with naCSF to get a resistance of 1–2 MΩ. Field potentials were recorded in CA1 stratum radiatum using a Ag/AgCl bridge with CV7B head stage (Molecular Devices, Sunnyvale, CA) located ~1–2 mm from the stimulating electrode. LTP was induced using a high frequency stimulation protocol (3 x 100 Hz, 20 seconds) as previously described (32).

To assess basal synaptic strength, 250 ms stimulus pulses were given at 10 intensity levels (range, 100–1000 mA) at a rate of 0.1 Hz. Three field potentials at each level were averaged, and measurements of fiber volley (FV) amplitude (in millivolts) and fEPSP slope (millivolts per millisecond) were performed using Clampfit 10.7 software. Synaptic strength curves were constructed by plotting fEPSP slope values against FV amplitudes for each stimulus level. Baseline recordings were obtained for 10 minutes by delivering single pulse stimulations at 20 second intervals. All data are represented as a percentage change from the initial average baseline fEPSP slope obtained for the 10 min prior to HFS. Three brain slices were recorded per animal and averaged to calculate the response per animal.

### Statistical analysis

Statistical tool package available on the BioRender web-based platform (Toronto, ON) or GraphPad Prism 10 (San Diego, USA) were used. For data exhibiting a normal distribution, a one-way Analysis of Variance (ANOVA) was performed, followed by Tukey’s multiple comparison test. When data were not normally distributed, the Kruskal-Wallis test with Dunn’s multiple comparison test were used. All data are presented as the mean ± standard deviation (SD). A p-value of less than 0.05 was established as the threshold for statistical significance.

## Results

### Changes in body weight and burn wound size after HMGB1 antibody treatment

**Figure 2:**
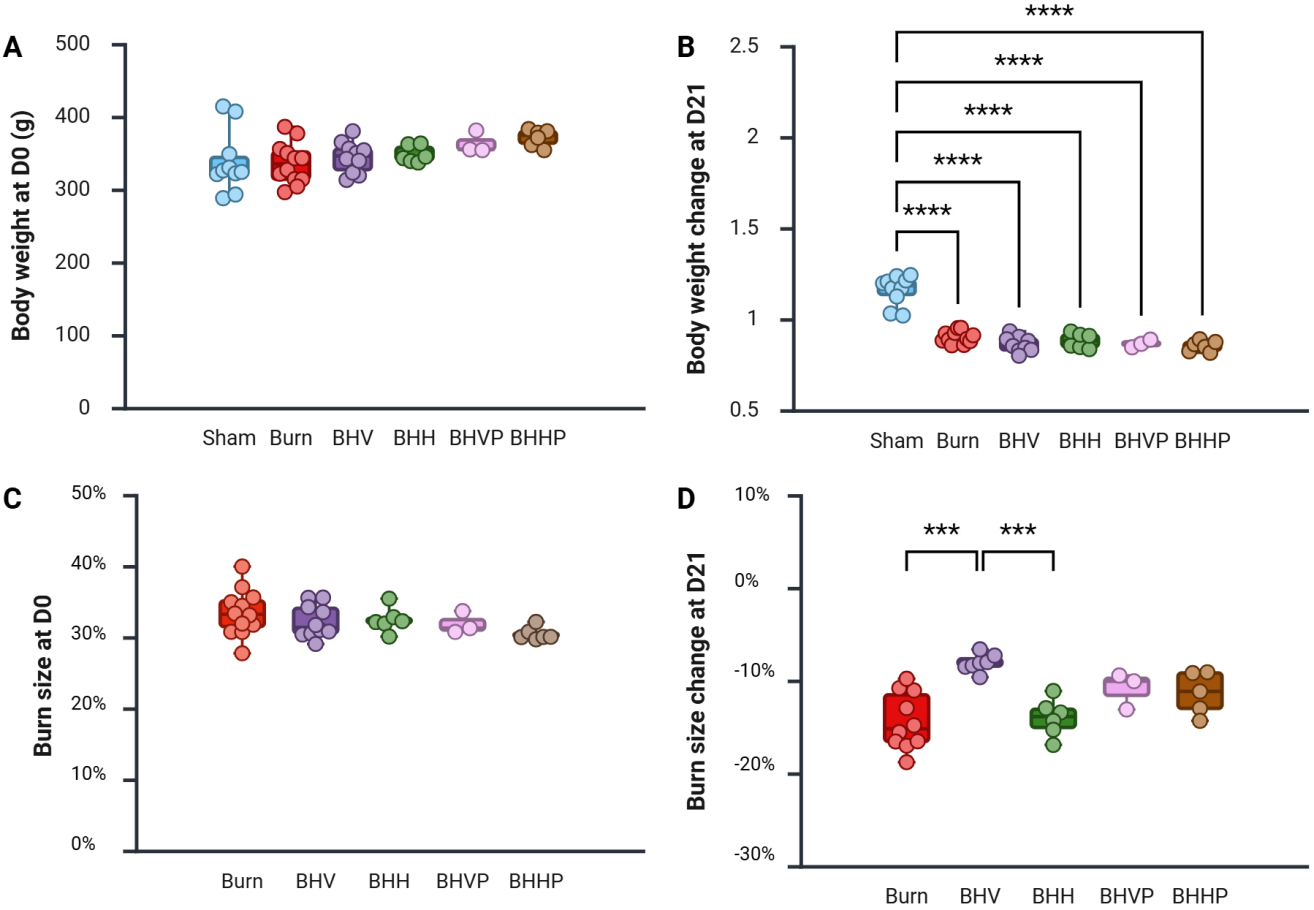
Changes in rat body weight and wound size post-burn injury. **(A)** Body weight recording among 6 groups at day 0 before procedure. **(B)** The ratio of individual rat body weight at day 21 vs. day 0. **(C)** Individual rat wound size at day 0 after receiving burn procedure. Individual burn size was normalized to total body surface area (TBSA). **(D)** Individual change in burn size was calculated as difference in TBSA burn size between day 21 and day 0. Sham (n=10) and Burn (n=9) without treatment. BHV (n=7) and BHVP (n=3) rats were burned, hindlimb unloaded, and treated with vehicle Ab by IP or IP/pump, respectively. BHH (n=6) and BHHP (n=6) rats were burned, hindlimb unloaded, and treated with anti-HMGB1 antibody by IP or IP/pump, respectively. Significance was calculated by one-way ANOVA with Tukey’s *post-hoc* test as data passed the Shapiro-Wilk normality test (***p<0.001, ****p<0.0001).

Following a 5-day acclimatization period in metabolic cages, 47 rats (mean body weight: 345.79 ± 28.52 g) were randomly assigned to six experimental groups before the experiment, ensuring no significant initial differences among them. (**Figure 2A**) At the beginning of the experiment, the burn size across the five groups that underwent the burn procedure also showed no significant differences (mean ± SD: 32.31 ± 2.50% TBSA). (**Figure 2C**) At the conclusion of the study period at 21 days post-burn, the ratios of final to initial body weight indicated that all burned animals experienced significant body weight loss compared to the sham group (p < 0.05). (**Figure 2B**) However, no significant difference in body weight change was observed among any of the burn groups (with or without vehicle or HMGB1 Ab treatment. Regarding wound healing, burn-injured rats subjected to 14 days of hindlimb unloading and vehicle treatment (BHV) exhibited reduced wound healing with less than a 10% change in wound size when compared to burn rats without hindlimb unloading that exhibited 14% decline in wound size from day 0 to day 21 post-injury (BHV: -8.09 ± 0.95% vs. Burn: -14.41 ± 3.05%, p < 0.05). Further, burn-injured rats with hindlimb unloading and treated with anti-HMGB1 antibody (BHH) demonstrated enhanced wound healing, evidenced by 14% reduction in wound size compared to those receiving vehicle antibody treatment (BHH: -14.03 ± 2.00% vs. BHV: -8.09 ± 0.95%, p < 0.05). No significant effect was observed when burn-injured rats with hindlimb unloading were treated with anti-HMGB1 or vehicle Ab using mini pump implantation (BHHP: -11.37 ± 2.31% vs. BHVP: -10.89 ± 1.96%),. (**Figure 2D**) These results suggested that 30% TBSA burn injury caused a significant body weight loss that was not further exacerbated by 14 days-immobilization and 7 days recovery period. Treatment with anti-HMGB1 Ab (intraperitoneal route) enhanced the wound healing in burn rats.

### Systemic immune profile in burn injury rats with and without anti-HMGB1 antibody treatment

**Figure 3:**
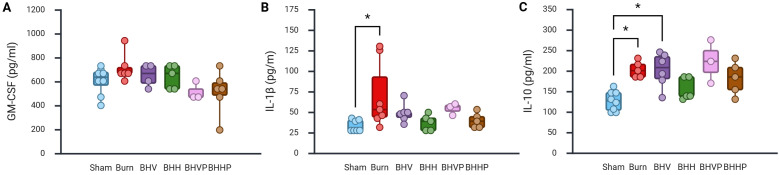
Systemic biomarkers of inflammation in burn rats with and without treatment. A panel of 10 cytokines was analyzed using the Milliplex MAP Rat Cytokine/Chemokine Magnetic Bead Panel. Shown are **(A)** GM-CSF, **(B)** IL-β, and **(C)** IL-10 levels. Data were collected from sham (n=8) and burn (n=6) rats without treatment; BHV (n=6) and BHVP (n=3) rats with burn/hindlimb unloading and vehicle Ab delivery by IP or IP/pump, respectively; and BHH (n=6) and BHHP (n=6) rats with burn/hindlimb unloading and anti-HMGB1 Ab delivery by IP or IP/pump, respectively. Statistical analysis was performed using a Kruskal-Wallis test with Dunn’s *post-hoc* test (*p<0.05).

Previously, proinflammatory cytokine response at the transcriptional level was observed in burn rats with or without immobilization (34). In this study, we observed that plasma levels of GM-CSF were not significantly changed among the studied groups. (**Figure 3A**) Interleukin-1 beta (IL-1β) and Interleukin-10 (IL-10) were significantly elevated at day 21 in the burn group compared to the sham group (Burn: 69.72 ± 40.57 pg/mL and 203.22 ± 19.74 pg/mL; Sham: 33.16 ± 7.06 pg/mL and 126.45 ± 24.03 pg/mL, respectively, p < 0.05) (**Figure 3B**). IL-1β was not significantly changed among the burn/immobilized groups, irrespective of the treatment. Plasma IL-10 levels were decreased in burn/immobilized groups treated with anti-HMGB1 Ab (BHH and BHHP) than those noted in burn/immobilized groups treated with vehicle Ab (BHV and BHVP). (**Figure 3C**).

**Figure 4:**
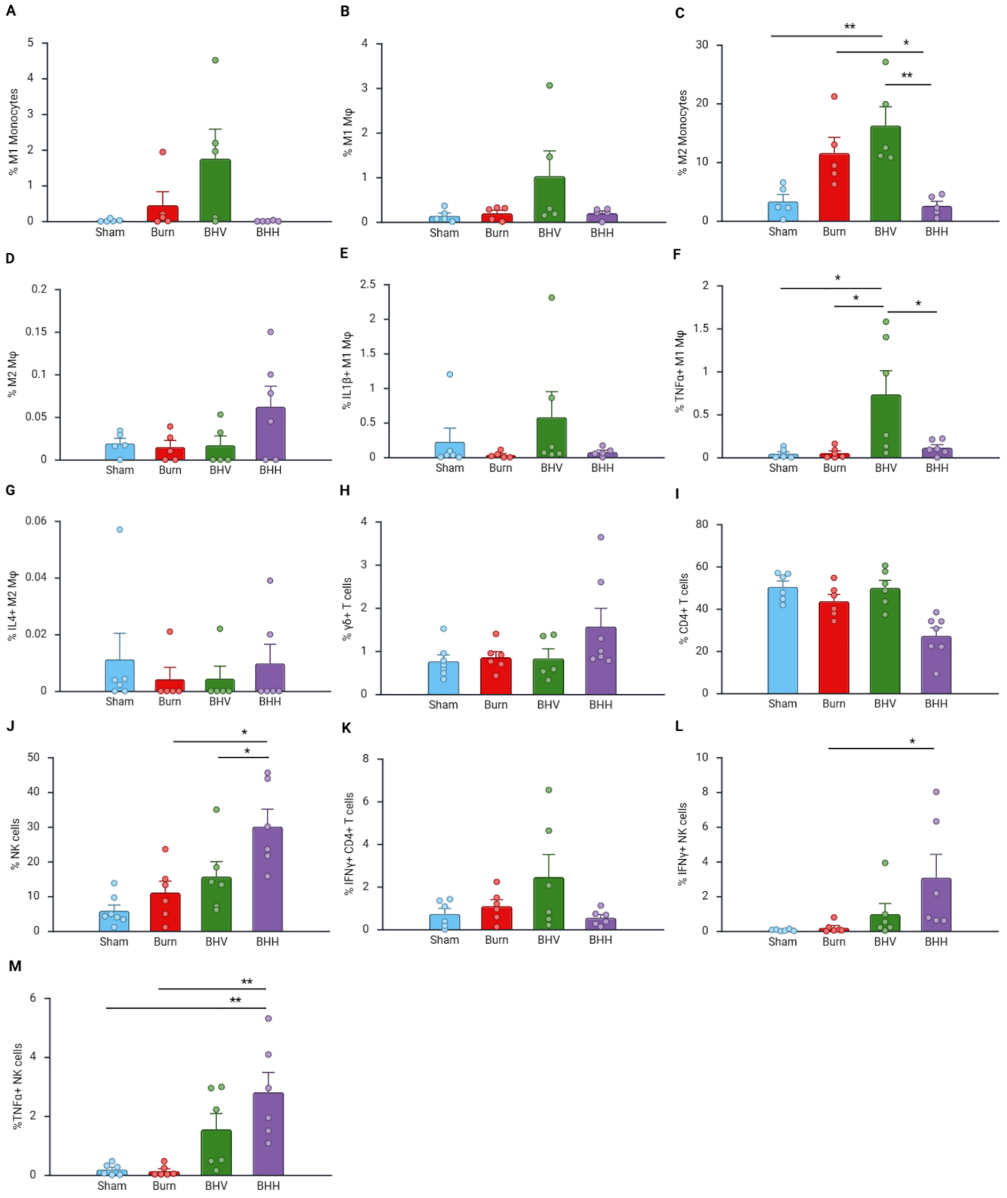
Effect of anti-HMGB1 treatment on immune cell profile in burn and hindlimb unloaded rats. Sprague Dawley rats were subjected to burn, immediately treated with one dose of anti-HMGB1 (BHH) or IgY control antibody (BHV) and hindlimb unloaded at 14 days. Animals were then released from tail suspension for 7-days mobile recovery before euthanasia. Sham and burn groups were pair-fed without any treatment. Single cell suspensions of peripheral blood mononuclear cells (PBMC) were labeled with fluorescent-conjugated antibodies and analyzed by flow cytometry. Percentages of **(A)** M1 monocyte, **(B)** M1 macrophages, **(C)** M2 monocytes, **(D)** M2 macrophages, **(E)** IL-1β+ M1 macrophages, **(F)** TNFα+ M1 macrophages, **(G)** IL4+ M2 macrophages, **(H)** gamma-delta T cells, **(I)** CD4+ T cells, **(J)** NK cells, **(K)** IFNγ+ CD4+T cells, **(L)** IFNγ+ NK cells, and **(M)** TNFα+ NK cells of all groups are shown. Data are derived from n ≥ 6 rats per group and plotted as mean values ± SEM. Significance was calculated by 1-way ANOVA/Tukey’s *post-hoc* test or Kruskal–Wallis H/Dunn’s *post-hoc* test. The p-values of ≤ 0.05 and ≤ 0.01 are plotted with * and ​ ​** respectively. Horizontal bar shows the compared groups.

Immune profiling of circulatory innate immune cells was performed using flow cytometry and following the gating strategy shown in **Supplementary Figure 1**. These data showed that the M1 (proinflammatory) monocytes and macrophages were increased in the PBMCs of burn/immobilized rats treated with vehicle only (BHV) and these subsets decreased to normal levels when burn/immobilized rats were treated with anti-HMGB1 Ab (BHH). (**Figures 4A, B**) At a functional level also, frequencies of IL-1β and tumor necrosis factor-alpha (TNF-α) producing M1 macrophages were decreased in HMGB1 Ab treated, BHH (vs. Burn only or BHV) group. (**Figures 4E, F**) Conversely, circulating M2 (anti-inflammatory) macrophages were increased in HMGB1 antibody treated, BHH (vs. Burn only or BHV) group (**Figures 4C, D, G**).

Circulating T cells were profiled by gating strategy shown in **Supplementary Figure 2**. These data showed a moderate increase in γδ TCR^+^ T cells capable of mounting MHC-independent, rapid immune response and a significant increase in fast acting, MHC lacking, innate, natural killer (NK) cells, while CD4^+^T cells that orchestrate the adaptive immune response were decreased in burn/immobilized rats given anti-HMGB1 antibody treatment (BHH vs. all other groups). (**Figures 4H–J**). At a functional level, while Interferon gamma (IFNγ) producing CD4^+^T cells were increased in burn/immobilized rats treated with vehicle only, IFNγ- and TNF-α expressing NK cells were increased in anti-HMGB1 treated burn/immobilized rats (vs. other groups). (**Figures 4K–M**).

These results suggested that treatment with HMGB1 Ab supported NK cells contributing to first line of immune defense and enhanced the anti-inflammatory/wound healing response by macrophages and γδT cells in burn/immobilized rats.

### HMGB1 antibody treatment rescues synaptic integrity affected by burn with hindlimb unloading by mitigating inflammation-induced hyperexcitability

**Figure 5:**
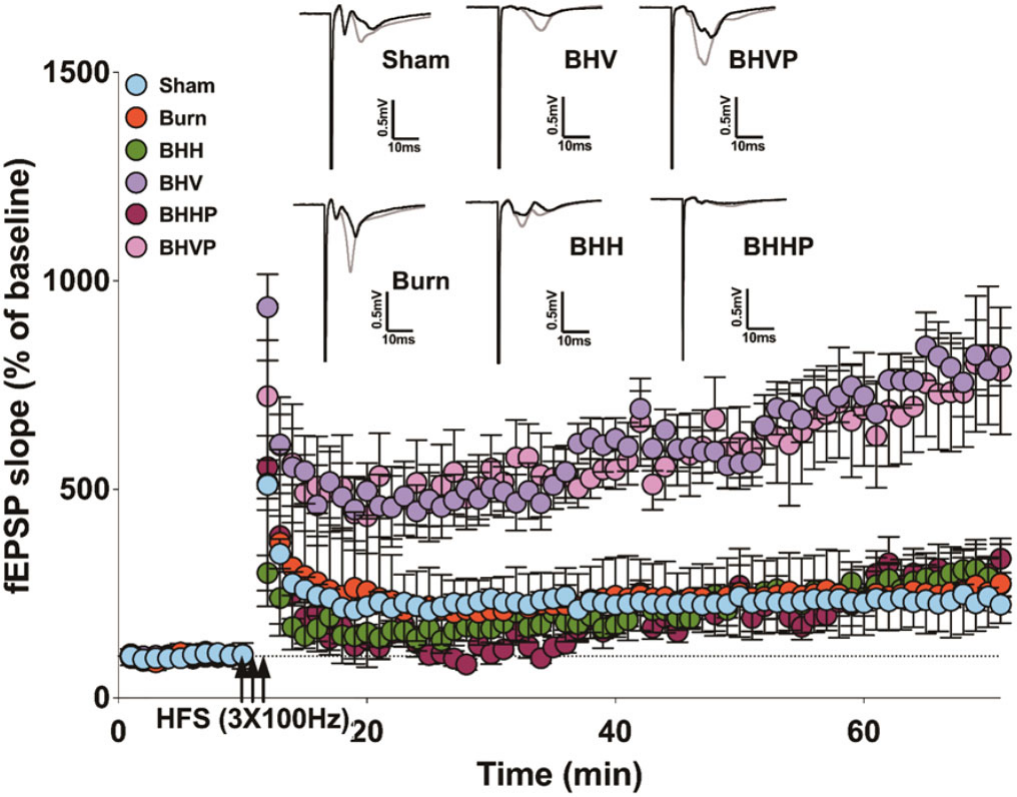
Recombinant HMGB1 treatment restores long-term potentiation at Schaffer collateral synapses following burn and hindlimb unloading. The field excitatory postsynaptic potential (fEPSP) slope is plotted as a percentage of the initial 10-minute baseline over 60 minutes total time where the effects of burn injury and hindlimb unloading, with or without HMGB1 treatment, on synaptic plasticity. The data shows that while the combined injury (burn and hindlimb unloading) impairs the maintenance of LTP, treatment with HMGB1, administered either directly or via a pump, rescues this deficit, restoring synaptic potentiation to levels comparable to sham controls. High-frequency stimulation (HFS; 3 trains of 100 Hz), indicated by the black arrows, was delivered at the 10-minute mark to induce LTP at the Schaffer collateral-CA1 synapses. Three slices were recorded for each animal and averaged. n represents the number of animals. Each data point represents the mean ± SEM for each experimental group. Sham (blue, n= 5), Burn (red, n= 5), BHV (purple, n=3, Burn and hindlimb unloaded rats treated with vehicle, BHH (green, n= 3, Burn and hindlimb unloaded rats treated with HMGB1), BHVP (pink, n=2, Burn and hindlimb unloaded rats with an implanted pump delivering vehicle) and BHHP (brown, n=3, Burn and hindlimb unloaded rats with an implanted pump delivering HMGB1 Ab). Inset panels show representative traces for each experimental group, providing a snapshot of synaptic strength before and after the induction of long-term potentiation (LTP). The traces visually correspond to the time-course data presented in the previous figures. Notably, the potentiation in animals subjected to burn injury appears qualitatively larger, potentially indicating a state of neuronal hyperexcitability. Each panel displays two overlaid fEPSP traces from a single representative slice. The black trace represents the average baseline synaptic response before HFS, and the grey trace represents the potentiated response 60 minutes after HFS. Calibration bars represent 0.5 mV and 10 ms.

**Figure 5** shows fEPSP slopes recorded over 60 minutes from samples across six groups, following 3 applications of 100Hz high frequency stimulation (HFS). Examination of the fundamental effects of thermal injury on hippocampal synaptic plasticity revealed that burn injury alone did not significantly alter the HFS-LTP profile at Schaffer collateral synapses. In both sham and burn groups, fEPSP slopes showed optimal potentiation. However, burn rats subjected to 14 days of immobilization and vehicle treatment (BHV and BHVP) exhibited a dramatic elevation of fEPSP slopes, exceeding the range of 500%. Notably, anti-HMGB1 antibody treatment significantly attenuated this elevation in burned and immoobilized rats, with similar effects observed across both delivery routes (BHH and BHHP). Relative to prior mouse synaptotoxicity paradigms (often decreased LTP), this model shows pathologic over−potentiation/hyperexcitability representing the versatility of using synaptic dysfunction as model that can demonstrate how overcompensation as well as underperformance can be quantified reproducibly.

**Figure 6:**
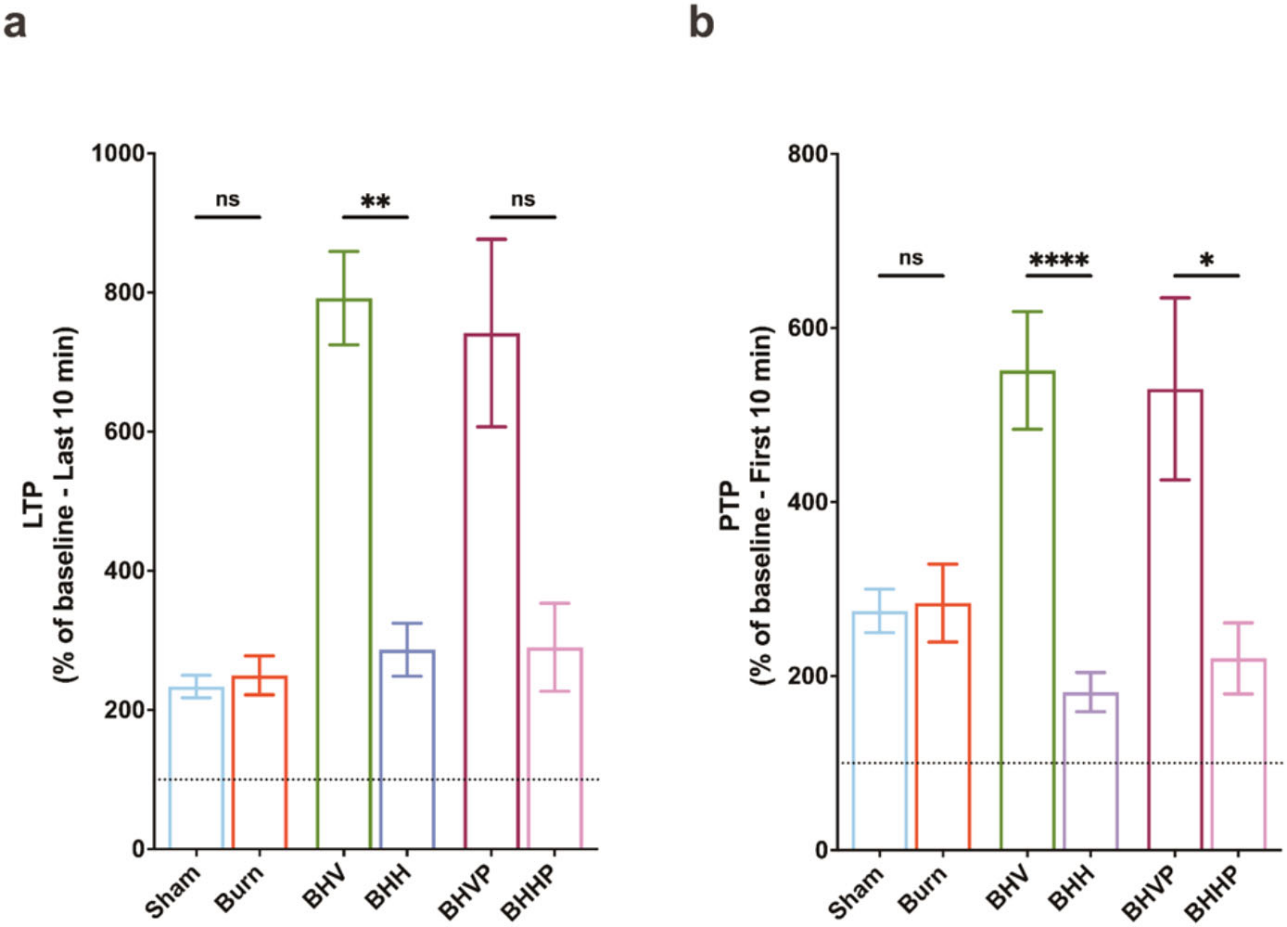
Potentiation quantification at Schaffer collateral synapses in hippocampus of burn rats with and without treatment. **(a)** Quantification of Long-Term Potentiation (LTP), measured as the average fEPSP slope during the final 10 minutes of recording (60–70 min post-HFS). **(b)** Quantification of Post-Tetanic Potentiation (PTP), measured as the average fEPSP slope during the first 10 minutes immediately following HFS (10–20 min). Three slices were recorded for each animal and averaged. n represents the number of animals. Each data point represents the mean ± SEM for each experimental group. Sham (blue, n= 5), Burn (red, n= 5), BHV (purple, n=3, Burn and hindlimb unloaded rats treated with vehicle, BHH (green, n= 3, Burn and hindlimb unloaded rats treated with HMGB1), BHVP (pink, n=2, Burn and hindlimb unloaded rats with an implanted pump delivering vehicle) and BHHP (brown, n=3, Burn and hindlimb unloaded rats with an implanted pump delivering HMGB1 Ab). Statistical analysis was performed using a Kruskal-Wallis test with uncorrected Dunn’s *post-hoc* test. Comparisons are shown between each treatment group and its respective vehicle control (i.e., BHH vs. BHV; BHHP vs. BHVP). Asterisks denote statistical significance (*p < 0.05, **p < 0.01, and ****p<0.0001).

Further time-course quantification analyses at the early post-tetanic potentiation (PTP) and the later long-term potentiation (LTP) were performed. These data showed that 14 days of immobilization with vehicle treatment induced an exaggerated PTP and LTP in burn-injured animals (compare BHV and BHVP with Burn group, p<0.05). (**Figures 6A, B**) Administration of anti-HMGB1 antibody via both routes regulated the initial PTP response and restored the stable LTP to normal levels in burn/immobilized rats (compare BHH and BHHP with BHV and BHVP groups, p<0.05). While both treatment delivery routes demonstrated similar initial effects, the significant difference in LTP maintenance was not sustained in the treatment group receiving pump implantation. (**Figure 6A**).

**Figure 7:**
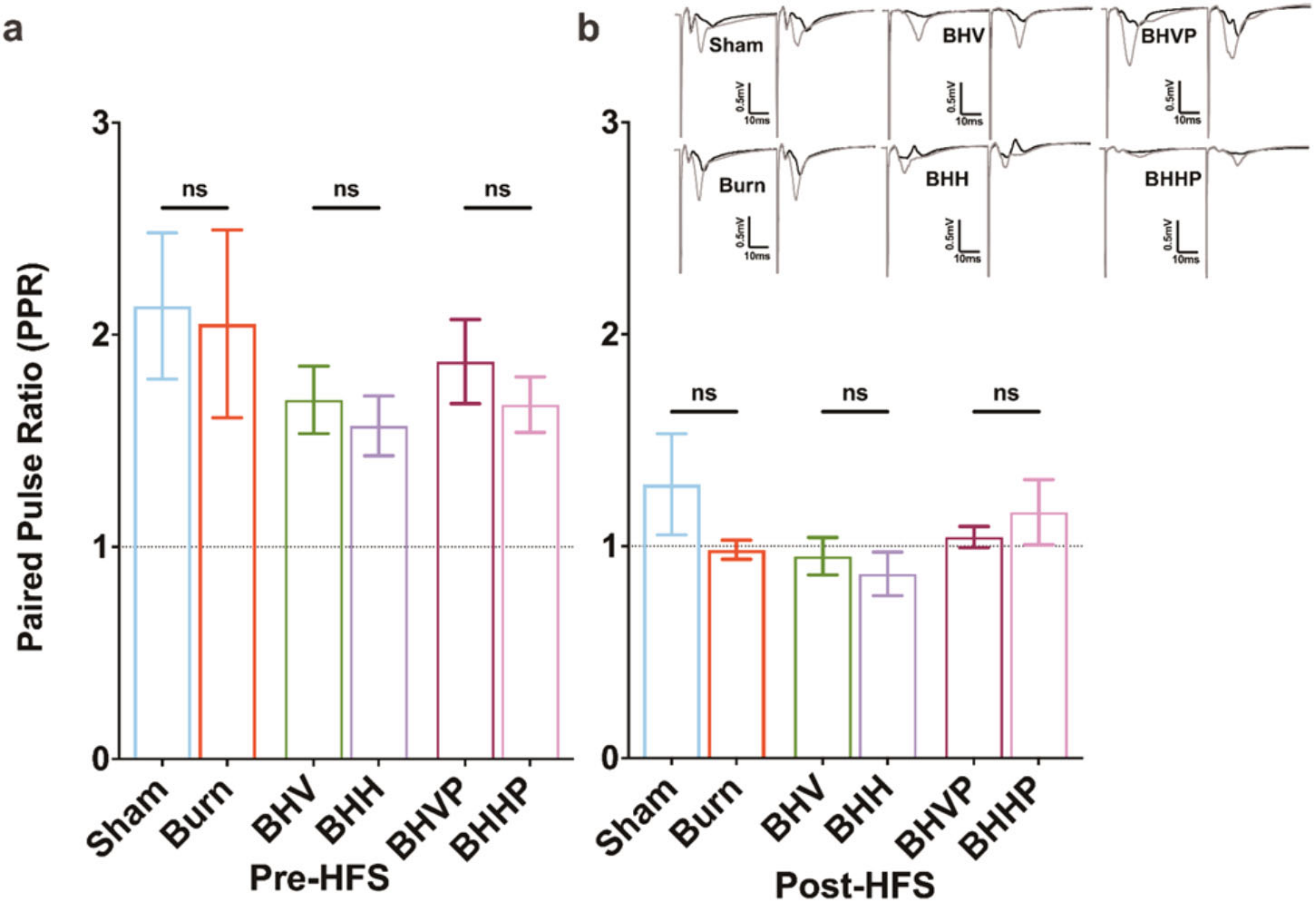
Paired-pulse facilitation ratio (PPR) analysis for presynaptic function estimation **(a)** Paired-pulse ratio measured before baseline, before the HFS-LTP induction protocol. **(b)** Paired-pulse ratio measured after the HFS-LTP induction protocol (after 60–70 minutes). Three slices were recorded for each animal and averaged. n represents the number of animals. Each data point represents the mean ± SEM for each experimental group. Sham (blue, n= 5), Burn (red, n= 5), BHV (purple, n=3, Burn and hindlimb unloaded rats treated with vehicle, BHH (green, n= 3, Burn and hindlimb unloaded rats treated with HMGB1), BHVP (pink, n=2, Burn and hindlimb unloaded rats with an implanted pump delivering vehicle) and BHHP (brown, n=3, Burn and hindlimb unloaded rats with an implanted pump delivering HMGB1 Ab). Inset panels show representative traces for each experimental group, providing a snapshot of fEPSP before and after the induction of LTP. Each panel displays two overlaid fEPSP traces from a single representative slice. The black trace represents the average baseline synaptic response before HFS, and the grey trace represents the potentiated response 60 minutes after HFS. Calibration bars represent 0.5 mV and 10 ms. Statistical analysis calculated by one-way ANOVA with Kruskal-Wallis *post-hoc* test revealed no significant differences between any of the groups in either condition.

Presynaptic function was assessed by measuring the paired-pulse facilitation ratio (PPR). These measurements were taken both before (pre) and after (post) high frequency stimulation. These data revealed no significant differences in PPR among the six experimental groups at baseline or after LTP induction. (**Figure 7**) PPR indicates release probability is not detectably altered; however, IO analysis suggests increased presynaptic axon excitability (FV–stimulus left-shift) in BHV/BHVP, pointing to altered excitability rather than transmitter release changes. Further, within-group analysis indicates that while HFS consistently induced a significant change in PPR (p < 0.05), such presynaptic response was uniform and comparable across all groups. (**Supplementary Figure 3**).

**Figure 8:**
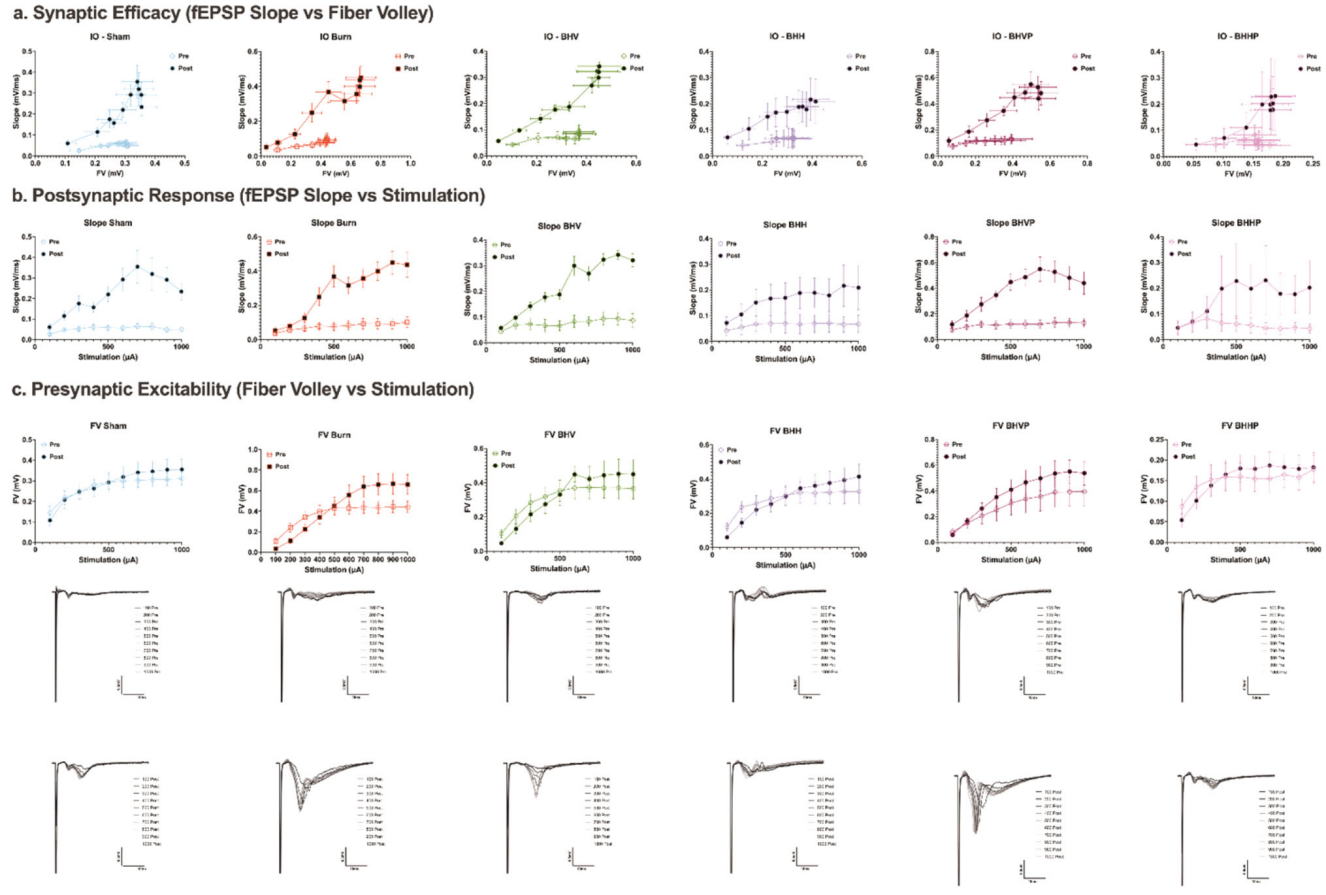
Input-output characteristics and synaptic potentiation profile of burn rats with and without HMGB1 Ab treatment. **(a)** Synaptic Efficacy (fEPSP Slope vs. Fiber Volley): representing the postsynaptic response (fEPSP slope, mV/ms) as a function of the presynaptic input (Fiber Volley amplitude, mV). Following HFS, the Post-HFS curve (filled circles) shifts upward and to the left relative to the pre-HFS curve (hollow circles). This “E-S potentiation” signifies a greater postsynaptic depolarization for a given presynaptic volley, a key characteristic of successful LTP. **(b)** Postsynaptic Response (fEPSP Slope vs. Stimulation): representing the fEPSP slope against the increasing stimulation intensity (µA). The post-HFS curve is left-shifted, indicating that a larger synaptic response is achieved at lower stimulation intensities after potentiation. **(c)** Presynaptic Excitability (Fiber Volley vs. Stimulation): representing the presynaptic fiber volley amplitude against stimulation intensity. The Pre- and Post-HFS curves are largely overlapping, confirming that the LTP induction protocol did not significantly alter the excitability of the presynaptic fibers. Three slices were recorded for each animal and averaged. n represents the number of animals. Each data point represents the mean ± SEM for each experimental group. Sham (blue, n= 5), Burn (red, n= 5), BHV (purple, n=3, Burn and hindlimb unloaded rats treated with vehicle, BHH (green, n= 3, Burn and hindlimb unloaded rats treated with HMGB1), BHVP (pink, n=2, Burn and hindlimb unloaded rats with an implanted pump delivering vehicle) and BHHP (brown, n=3, Burn and hindlimb unloaded rats with an implanted pump delivering HMGB1 Ab). Panels below each group after **(c)** show representative traces for each experimental group, where the top panel is pre-HFS and the bottom panel is post-HFS providing a snapshot of fEPSP before and after the induction of LTP. Calibration bars represent 0.5 mV and 10 ms.

The input-output (IO) characteristics, which delineate the relationship between synaptic input strength and the resultant neuronal response, including synaptic efficacy, postsynaptic response, and presynaptic excitability, were individually assessed for all six experimental groups (**Figure 8**). In sham group, fEPSP slope (Slope, mV/ms) was evaluated as a function of the presynaptic input (Fiber Volley amplitude or FV in mV). Post-HFS curve demonstrated an upward and leftward shift relative to the pre-HFS curve. This “E-S potentiation (EPSP-to Spike potentiation)” indicates an augmented postsynaptic depolarization for a given presynaptic volley, a hallmark of successful LTP. When plotting the fEPSP slope against increasing stimulation intensity (µA), the post-HFS curve was left-shifted, signifying a greater synaptic response at lower stimulation intensities post-potentiation. Assessment of presynaptic excitability (FV vs. Slope) revealed largely overlapping pre- and post-HFS curves, confirming that the LTP induction protocol did not significantly alter presynaptic fiber excitability.

Burn alone produced IO and LTP profiles comparable to sham (no evidence of basal hyperexcitability), although a trend toward greater potentiation was observed. The burn with hindlimb unloading (BHV) group exhibited a more intricate response to HFS. While potentiation was evident, its relationship to presynaptic input was altered, with a significant component of the potentiation appearing to stem from increased presynaptic fiber excitability. The coupling between pre- and postsynaptic elements was disrupted compared to the sham group. The post-HFS curve showed potentiation at lower fiber volley values but failed to maintain a clear separation from the pre-HFS curve at higher inputs, suggesting that E-S potentiation was blunted or unstable under this condition. When analyzed against stimulation intensity, clear potentiation was observed, with the post-HFS curve left-shifted relative to the pre-HFS curve, indicating an overall increase in synaptic output post-HFS. A critical distinction from the sham group was noted in presynaptic excitability, where the post-HFS curve was markedly left-shifted compared to the pre-HFS curve, signifying a substantial increase in presynaptic axon excitability following the HFS protocol.

In burn with hindlimb unloading and anti-HMGB1 Treatment (BHH) group, anti-HMGB1 treatment successfully restored a classic potentiation profile. The post-HFS curve displayed a distinct upward and leftward shift relative to the pre-HFS curve, indicating a recovery of normal E-S potentiation and ameliorating the deficit observed in the BHV group. A robust left-shift in the post-HFS curve for the postsynaptic response confirmed that the capacity for strong LTP was restored by HMGB1 treatment. Crucially, the aberrant increase in presynaptic excitability observed in the BHV group was completely prevented by HMGB1. The pre- and post-HFS curves were largely overlapping, mirroring the stable presynaptic function of the sham controls and indicating a comprehensive rescue of synaptic plasticity mechanisms.

Burn with hindlimb unloading and vehicle pump (BHVP) group, consistent with the other vehicle-treated group (BHV), displayed a potentiation profile distinct from sham controls, primarily characterized by an increase in presynaptic fiber excitability (FV) following HFS. The post-HFS curve for synaptic efficacy was shifted upward and to the left of the pre-HFS curve, indicating the presence of E-S potentiation. Strong potentiation was evident from the pronounced left-shift of the post-HFS curve for the postsynaptic response, signifying a robust increase in overall synaptic output after HFS. Like the BHV group, in the BHVP group, the post-HFS curve was markedly left-shifted compared to the pre-HFS curve for presynaptic excitability, demonstrating a significant increase in presynaptic axon excitability induced by the HFS protocol—a key pathological feature of the combined injury model not observed in sham animals.

In burn with hindlimb unloading and anti-HMGB1 pump (BHHP) group, continuous anti-HMGB1 treatment via pump implantation also resulted in a complete rescue of synaptic function. The potentiation profile of the BHHP group was nearly identical to that of sham controls and demonstrated a full reversal of the pathologies observed in its corresponding vehicle-pump group (BHVP). For synaptic efficacy, the post-HFS curve displayed a classic upward and leftward shift, indicating a full restoration of normal E-S potentiation. A robust left-shift in the post-HFS curve for the postsynaptic response confirmed that the capacity for strong LTP, like that in sham animals, was restored by the continuous HMGB1 treatment. Critically, the HFS-induced increase in presynaptic excitability seen in the vehicle control group was completely absent, with the pre- and post-HFS curves largely overlapping. This demonstrates that continuous HMGB1 treatment normalized presynaptic function, restoring the stable profile seen in sham controls.

## Discussion

### Main findings

Severe burns frequently result in long-term cognitive deficits and psychological morbidities (35). Extensive evidence suggests that acute neuronal damage, often caused by infiltrating immune cells, plays a significant role in various trauma and inflammatory conditions (10) (11) (12).^,,^ Following burn injuries, consistent systemic inflammatory responses are linked to local tissue immune cell infiltration (25, 36). While previous research has documented burn-induced nervous system impairments, including spinal cord pathology (37) and peripheral neuropathy (38), our study uniquely demonstrates CNS synaptic dysfunction in a clinically relevant model of severe burn combined with 14 days of immobilization.

Our key findings include significant alterations in hippocampal long-term potentiation (LTP) and a distinct systemic immune/inflammatory profile following severe burn and hind-limb unloading. Crucially, we found that early blockade of HMGB1 with a neutralizing antibody not only dampened the systemic immune profile and inflammation response but also preserved the neural synaptic function. These results collectively suggest a promising therapeutic opportunity where targeting HMGB1 shortly after injury could modulate burn-driven immune responses and help protect hippocampal circuits from long-term functional loss. A novel aspect of our study is that synaptic dysfunction after systemic trauma is not solely characterized by reduced LTP. Burn with prolonged immobilization produced persistent hippocampal hyperexcitability and altered interneuron coupling, and early systemic HMGB1 neutralization normalized this phenotype. This suggests that a peripheral DAMP signal can lead to long-lasting circuit-level plasticity changes.

### Synaptic function mechanism and examination in the hippocampus

In this model, synaptic dysfunction primarily manifested as pathologic hyperexcitability/exaggerated potentiation rather than reduced LTP. We therefore interpret ‘rescue’ by anti-HMGB1 antibody as normalization of potentiation magnitude and IO coupling, not simply restoration of a diminished response.

The Schaffer collateral pathway is a major hippocampal projection originating from the axons of CA3 pyramidal neurons. These axons give rise to extensive collateral branches that deliver a primary excitatory input to CA1 pyramidal neurons. This connection represents a core component of the hippocampal tri-synaptic circuit, which is essential for the processing and consolidation of new memories. The Schaffer collaterals also serve as a foundational model for examining synaptic plasticity, including LTP and long−term depression (LTD), enduring modifications in synaptic strength that are widely regarded as fundamental cellular mechanisms of learning and memory. Glutamatergic signaling through the CA3–CA1 Schaffer collateral pathway is essential for hippocampal functions such as spatial navigation and episodic memory (39). In Alzheimer’s disease, β−amyloid oligomers disrupt NMDA−receptor trafficking and Ca²^+^ signaling, impairing CA1 LTP in proportion to memory loss (40). In major depressive disorder, chronic glucocorticoid exposure reduces AMPA/NMDA receptor expression and BDNF in CA1, suppressing LTP, while antidepressant treatment restores both (41). Aging also demonstrates diminished LTP induction and its maintenance in the CA3–CA1 circuit and is associated with reduced CAMKII activation resulting in altered postsynaptic scaffolding proteins (42).

Drawing parallels with similar disease models (43) (32),, we observed synaptic dysfunction characterized by overexcitability, potentially attributed to neuroinflammation profiles. It is crucial to acknowledge that differences in directionality may be influenced by factors such as injury type (systemic inflammatory trauma versus proteinopathy), species, and timing (21 days post-injury). Consequently, consolidated data for burn injuries at 21 days post-injury is often limited, which is understandable given that the most significant inflammatory surge typically diminishes by the time. Unlike many mouse synaptotoxicity studies where altered release probability contributes to LTP deficits, we found that PPR remained unchanged, suggesting that transmitter release probability is preserved. Instead, the pathology seems to involve altered axonal recruitment and excitability (FV–stimulus left-shift) and postsynaptic E–S coupling. Notably, we observed a significant elevation in post-synaptic function impairment 21 days after the injury, particularly following a 14-day period of immobilization.

Extended bedrest or prolonged immobilization is well-documented to impair both peripheral neuromuscular and central neural function (29), often involving disruptions to synapse-related mechanisms (44). We know that prolonged immobilization leads to skeletal muscle atrophy (45), which in turn could create a negative feedback loop affecting nerve-to-muscle excitability. Given that burn-induced inflammation typically subsides and animals recover from immobilization after seven days, one might expect a relatively swift recovery of pre-synaptic function as the negative feedback signals subside. However, what we surprisingly observed was a clear and persistent presence of abnormal post-synaptic function. This suggests that while some aspects might recover, the post-synaptic side remains significantly affected.

Post-synaptic function can be significantly impaired by several factors, such as neuroinflammation, oxidative stress, nutrition, mechanical shift etc. Our hindlimb unloading model was originally designed for NASA and was used for astronaut metabolic study under microgravity (46). We previously found that 14 days immobilization activated oxidative stress pathway while burn injury sustained an inflammation response at the transcriptional level (34). Long-term exposure to simulated microgravity, which mimics the unloading effects of bed rest, has been shown to negatively impact hippocampal synaptic function.

While our current study did not primarily focus on animal cognitive changes, a separate pilot investigation yielded some interesting preliminary findings. In that study, 18 animals were grouped with the same setting treatment as in the current study. (**Supplementary Figure 4**) The burn/immobilized group exhibited stress and fear behaviors with decreased locomotive activities and increased corner stay at three months post-injury. Encouragingly, these behavioral deficits were alleviated with HMGB1 antibody treatment. Furthermore, even with a small cohort of animals, we observed that cytokine changes at the three-month mark continued to show patterns like those seen at 21 days after the initial injury. As open field test is not a direct cognitive test, further specific cognitive tests will be implanted, including Y-maze/Barnes maze for spatial learning, Crawley’s Three Chamber test for sociability, Marble Burying test for repetitive behavior, and Contextual Fear Conditioning for memory performance.

### CNS synaptic function related to immune profile/inflammation responses

Severe burn injuries can trigger significant inflammation and alter the immune cell profile. Previous research consistently highlights the crucial role of immune cells and inflammatory responses over time following burn. Typically, inflammatory responses are initiated within 24 hours (47), with a broad activation of immune cells observed in the blood by day 3 (25). HMGB1, in particular, is known to be a key regulator of immune cell activity, influencing the subsequent tissue response (22). We previously showed that a complex interplay of pro- and anti-inflammatory cytokines fluctuated for up to 14 days post-injury in rat models (36). In this study, we screened a panel of cytokines including GM-CSF, IFN-γ, IL-1α, IL-1β, IL-2, IL-4, IL-6, IL-10, IL-12(p70) and TNF-α, yet only a subset was consistently detected. Interestingly, we observed that IL-1β, a potent pro-inflammatory cytokine produced by immune cells like macrophages, remained elevated for up to 21 days post-burn. Conversely, IL-10, a crucial anti-inflammatory protein vital for regulating immune responses and promoting tissue healing, was elevated in both the burn and burn with hindlimb unloading (BHV) groups at day 21. This elevation of IL-10 suggests ongoing tissue healing, following the delayed impact of 14 days of immobilization.

### Clinical setting of burn/immobilization

Current burn/immobilization model is specifically designed to mimic clinical settings, allowing us to investigate how patients recover from severe injuries, particularly when they experience unavoidable, extended periods of immobilization during an ICU or hospital stay. These models are already well-established for evaluating both clinical pharmacotherapeutic treatments and exercise interventions (30, 48, 49). In this study, our primary objective was to investigate the potential therapeutic benefits of systemically reducing HMGB1 early after a burn injury, following a scenario that closely mirrors clinical practice. Interestingly, while previous research has highlighted burn-induced inflammation as a primary driver, we observed minimal inflammation and immunity profile changes in our animals. This is not entirely surprising, however, because unlike previous studies that examined animals immediately after 14 days of hindlimb unloading, we allowed our animals an additional seven days of mobile recovery. During this mobile period, the animals demonstrated more compensatory responses, which likely contributed to moderate differences in inflammatory and immune profiles.

### Anti-HMGB1 treatment in the study

Given that anti-chicken polyclonal IgY antibodies typically have a short biological half-life of only 1 to 2 days in rodents (50), we devised a strategy to extend their effective therapeutic window. We split the total dose into two, with one portion administered via a subcutaneous mini-pump designed to release the antibody consistently over a 7-day period, and rest was given intraperitoneally in one dose (BHHP). Therefore, we could keep the total dose same as another treatment group (BHH). When comparing animals with and without the osmotic pump implantation, we considered potential physical restrictions from the implantation anatomy. We observed increased irritation in several animals at the end of the hindlimb suspension period, which could potentially influence outcomes like wound healing and inflammatory cytokine profiles. However, despite this, we didn’t find any significant alterations in food intake between the groups with and without pump implantation.

Based on the information currently available, we speculate that the observed effects are primarily focused on acutely modulating the innate immune response following burn. It is also possible that the dose administered was on the higher side for the desired treatment effect. Since we have not tested certain parameters (e.g., a full dose-response curve), these aspects warrant further investigation in future studies.

Regarding general animal welfare, it is well established that burn injuries typically lead to reduced food intake around 2–3 days post-injury, and this effect is often worsened by immobilization (51). We observed similar changes in our study, which we estimated by monitoring daily food supplementation and excretion from metabolic cages. While the anatomical pump implantation could theoretically interfere with feeding behavior, we ultimately did not detect significant changes in food or water intake across our different treatment groups.

### Clinical impact

Our study offers significant clinical insights, especially concerning burn patients. Early mobilization restrictions and bed rest may help reduce the hypermetabolic response, stabilize the patient’s pain, and lower the risk of skin−graft failure. However, longer hospital stays are consistently associated with poorer outcomes. In this study, we found that burn wound healing was delayed in burn rats with 14-day hindlimb unloaded, indicating that destructive impact of wound healing in burn care management. Further, we observed that prolonged immobilization dramatically worsens neural functional impairment in pre-clinical settings. It is particularly important to note that this impairment did not fully resolve even after seven days of ambulation recovery, which strongly suggests that additional exercise or other therapeutic interventions might be crucial to address such persistent effects. In previous study, we found that hindlimb unloading increases oxidative−stress pathways and accelerates muscle atrophy in burn rats, whereas resistance exercise attenuates burn− and immobilization−induced inflammation and oxidative stress signal pathways, thereby supporting recovery of muscle mass and function (34). Exercise even promotes its effect combined with other therapeutic agents, such as oxandrolone and insulin (30) (48).

Regarding the therapeutic agent tested in the study, the fact that chicken IgY antibodies do not possess an Fc region that interacts with mammalian Fc receptors is a notable point. This characteristic could potentially simplify or accelerate their development in clinical trials, possibly by influencing their immunogenicity or clearance mechanisms in human subjects. Overall, these findings are quite encouraging for trauma and burn patients. They underscore the potential benefits of prompt intervention in reducing the likelihood of long-term cognitive dysfunction. However, evaluating the optimal therapeutic window for such interventions needs further investigation, drawing upon insights from various studies and the evolving immune response profiles following burn injury.

### Limitation and future directions

While our study has yielded valuable insights, it also highlights several key areas for future exploration. We plan to enhance our examination methods by incorporating our well-optimized chemical LTP assessment technique - Fluorescence Assisted Single Synaptosome Long Term Potentiation (FASS-LTP) (52). We assess the glutamatergic synaptic integrity in different brain regions in the same animal using crude synaptoneurosomes and flow cytometry for a more profound understanding. Upon successful completion, we can provide a complete picture of how synaptic plasticity is similar or different across the different brain regions following burn injuries. Although morphological changes in burn pathology was not a primary focus due to experimental limitations, we recognize their importance and aim to examine them concurrently in future work, as structural and functional changes, while not always directly correlated, offer complementary information. Since our present study focused on a therapeutic functional approach, leaving the precise underlying mechanisms as a significant area for further elucidation, our future assessments will precisely differentiate the effects of anti-HMGB1 treatment on pure immobilization versus burn-induced effects.

We did not observe significant differences among groups, likely because the early post−burn surge in HMGB1 was no longer detectable at this later time point (see **Supplementary Figure 5**). Post−burn neuroinflammation will be characterized using NGS and IHC approaches to identify key molecular mediators, along with analysis of downstream RAGE/TLR/NF−κB signaling pathways, to further confirm the role of HMGB1 in regulating neuroinflammatory responses. While neural inflammation is widely accepted as a primary pathway leading to synaptic dysfunction following burn injury, our findings further highlight that prolonged immobilization significantly exacerbates this neural impairment. Though we demonstrated the systemic inflammation markers altered in burn and hindlimb unloaded rats with HMGB1 treatment, future study needs to further explore the direct evidence of neuroinflammation in CNS. Interestingly, our previous work indicated an overlapping and aggravated blood clot pathway in both burn and hindlimb unloading conditions. This suggests that the blood clot pathway could represent an additional, novel mechanism to explore in the interplay between HMGB1, burn injury, and hindlimb unloading.

## Conclusions

We have explored the therapeutic potential of targeting HMGB1 in preserving neural function. We demonstrated that extended immobilization dramatically worsened the synaptic function, which might be associated with subsequent cognitive impairment. This research highlights the significant potential of HMGB1 modulation and represents a promising first step in developing interventions for these complex conditions.

## Data Availability

The original contributions presented in the study are included in the article/**Supplementary Material**. Further inquiries can be directed to the corresponding authors.

## Supplementary material

Supplementary Figure 1 Gating strategy for innate immune cells by flow cytometry. PBMCs from all groups of rats were stained with 10-color fluorochromes, and innate immune cells were captured from acquired cells using a LSRII Fortessa. Briefly, after gating for live cells in forward and side scatter **(A-D)**, cell aggregates/doublets were ignored and live, CD45^+^ single leukocytes were gated for CD11b/c and MHCII expression **(E-F)**. The CD45+ CD11b+ MHCII+ cells were characterized as CD86+ (M1) and CD163+ (M2) macrophages (**G-H**). The MHCII- His48^hi^ (M1) and MHCII- His48^low^ (M2) monocytes were gated (**I-J**). CD11b+His48+ neutrophils were identified based on RP1+ phenotype (**K-O**). Each subset was further analyzed for the expression levels of proinflammatory and anti-inflammatory cytokines.

Supplementary Figure 2 Flow cytometry gating strategy for profiling the T cell subsets. PBMCs from all groups of rats were stained with 10-color fluorochromes, and lymphocytes were captured from acquired cells using a BD LSRII Fortessa flow cytometer. After gating for live lymphocytes in forward and side scatter (**A-C**), cell aggregates/doublets were ignored. The single live cells (**D**) were gated for α/β TCR (**E)**, and α/β TCR- γ/δ TCR+, α/β TCR- NK1.1+, and α/β TCR+CD4+T cell subsets (**F-H**) identified. Each subset was analyzed for the expression levels of various cytokines.

Supplementary Figure 3 A within-group analysis of the paired-pulse ratio (PPR) to assess the presynaptic properties. The bar graph displays the paired-pulse ratio (50ms interval), comparing the measurement taken before HFS (Pre) and after HFS (Post) for each experimental group. Bars represent the mean ± SEM. The groups are paired as follows: Sham (Pre: clear bar; Post: grey bar with blue borders, n=5); Burn (Pre: clear bar; Post: grey bar with red borders, n=5); BHV (Pre: clear bar; Post: grey bar with green borders bar, n=3, burn/hindlimb unloaded and treated with vehicle Ab intraperitoneally); BHH (Pre: clear bar; Post: grey bar with purple border, n=3, burn/hindlimb unloaded and treated with anti-HMGB1 Ab intraperitoneally); BHVP (Pre: clear bar; Post: grey bar with brown border, n=2, burn/hindlimb unloaded, and treated with vehicle Ab by IP/pump); BHHP (Pre: clear bar; Post: grey bar with pink border, n=3, burn/hindlimb unloaded, and treated with anti-HMGB1 Ab by IP/pump). A mixed-effects model (REML) was used to compare pre- and post-HFS values. The analysis showed a significant effect of HFS on the PPR, but this effect was comparable across all treatment groups, indicating that the presynaptic machinery responds similarly to LTP induction regardless of the experimental condition.

Supplementary Figure 4 Cognitive impairment and inflammation in burn rats. Eighteen rats were randomly grouped into sham (n=4), Burn (n=4), Burn/Hind-limb unloading/vehicle only (BHV, n=5) and Burn/Hind-limb unloading/HMGB1 Ab treatment (BHH, n=5) groups. (A-C) Cognitive impairment. Open field test was conducted on 4 consecutive days at 3 months after burn. All test data were pooled into each group. A) Total Locomotor Activity (TLMA) was calculated as the total distance traveled along 60 minutes of experiment period. B) The ratio of time spent in the center vs. periphery space (CenT/PerT), and C) The frequency of standing (Rearing) was calculated for animal anxiety and stress estimation. (D-E) Plasma levels of IL-1β and IL-10 are shown. Statistical significance was analyzed by one-way ANOVA with Tukey’s multiple comparisons test (*p<0.05, **p<0.01). Color codes in BioRender: Blue #6CC3F4; Red #E40D0D; Purple #825CA6; Green #378523; Pink #EEAAEE; Brown #A05208.

Supplementary Figure 5 Cognitive impairment and inflammation in burn rats. Plasma HMGB1 level in burn and hindlimb unloaded rats with anti-HMGB1 treatment. Data were collected from sham (n = 8) and burn (n = 6) rats without treatment; BHV rats (n = 6) with burn/hindlimb unloading and vehicle antibody delivered intraperitoneally; and BHH (n = 6) and BHHP (n = 6) rats with burn/hindlimb unloading and anti HMGB1 antibody delivered intraperitoneally. An additional 2–3 animals per group were excluded due to insufficient sample volume or outlier measurements.

## Glossary

HMGB1: High mobility group box 1
TBSA: Total body surface area
HLU: Hindlimb unloading
LTP: Long-term potentiation
PTSD: Post−traumatic stress disorder
CNS: Central nervous system
PAMPs: Pathogen−associated molecular patterns
DAMPs: Damage−associated molecular patterns
IACUC: Institutional Animal Care and Use Committee
NIH: National Institutes of Health
ARRIVE: Animal Research: Reporting of *In Vivo* Experiments
aCSF: Artificial cerebrospinal fluid
fEPSP: Field excitatory post-synaptic potential
HFS: High frequency stimulation
PTP: Post-tetanic potentiation
LTP: Long-term potentiation
PPR/PPF: paired-pulse facilitation ratio
IO: Input-output
FV: Fiber volley
EPSP: Excitatory postsynaptic potential
E-S potentiation: to Spike
BHV: Burn and hindlimb unloaded with vehicle treatment
BHH: Burn and hindlimb unloaded with anti-HMGB1 antibody treatment
BHVP: Burn and hindlimb unloaded with vehicle treatment delivered by mini pump
BHHP: Burn and hindlimb unloaded with anti-HMGB1 antibody treatment delivered by mini pump
IL-1β: Interleukin-1 beta
IL-10: Interleukin-10.
