# Supplementary material for: Immediate post-injury HMGB1 neutralization prevents synaptic dysfunction in burn and hindlimb unloaded rats
Source: Front Immunol. 2026 Jun 23;17:1867953. doi: 10.3389/fimmu.2026.1867953 (PMC13337465; doi:10.3389/fimmu.2026.1867953)
Supplement: Supplementary Figure 1 — Gating strategy for innate immune cells by flow cytometry. PBMCs from all groups of rats were stained with 10-color fluorochromes, and innate immune cells were captured from acquired cells using a LSRII Fortessa. Briefly, after gating for live cells in forward and side scatter (A-D), cell aggregates/doublets were ignored and live, CD45+ single leukocytes were gated for CD11b/c and MHCII expression (E-F). The CD45+ CD11b+ MHCII+ cells were characterized as CD86+ (M1) and CD163+ (M2) macrophages (G-H). The MHCII- His48hi (M1) and MHCII- His48low (M2) monocytes were gated (I-J). CD11b+His48+ neutrophils were identified based on RP1+ phenotype (K-O). Each subset was further analyzed for the expression levels of proinflammatory and anti-inflammatory cytokines. [file Presentation1.pptx]

## Slide 1
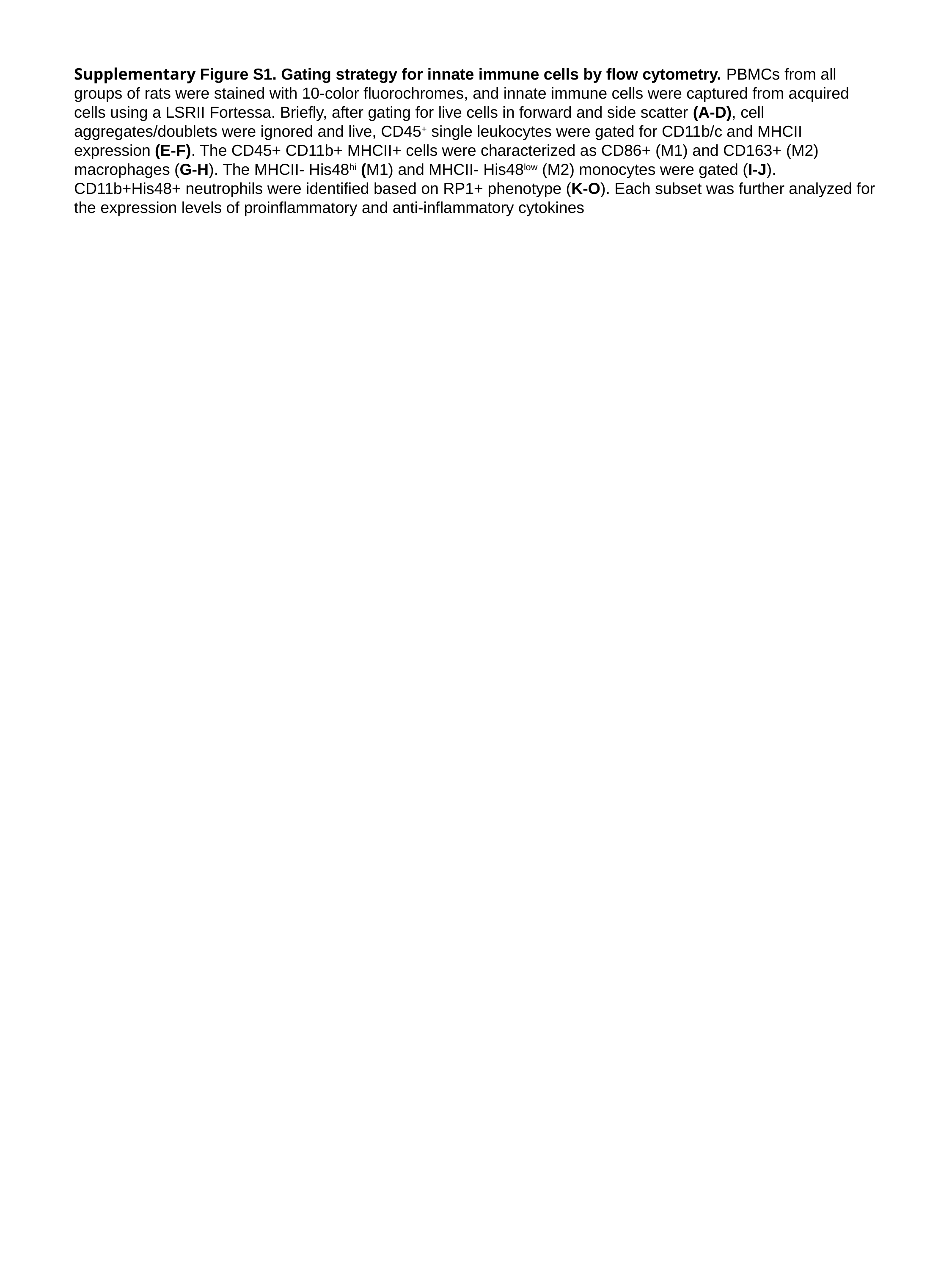

Supplementary Figure S1. Gating strategy for innate immune cells by flow cytometry. PBMCs from all groups of rats were stained with 10-color fluorochromes, and innate immune cells were captured from acquired cells using a LSRII Fortessa. Briefly, after gating for live cells in forward and side scatter (A-D), cell aggregates/doublets were ignored and live, CD45+ single leukocytes were gated for CD11b/c and MHCII expression (E-F). The CD45+ CD11b+ MHCII+ cells were characterized as CD86+ (M1) and CD163+ (M2) macrophages (G-H). The MHCII- His48hi (M1) and MHCII- His48low (M2) monocytes were gated (I-J). CD11b+His48+ neutrophils were identified based on RP1+ phenotype (K-O). Each subset was further analyzed for the expression levels of proinflammatory and anti-inflammatory cytokines

## Slide 2
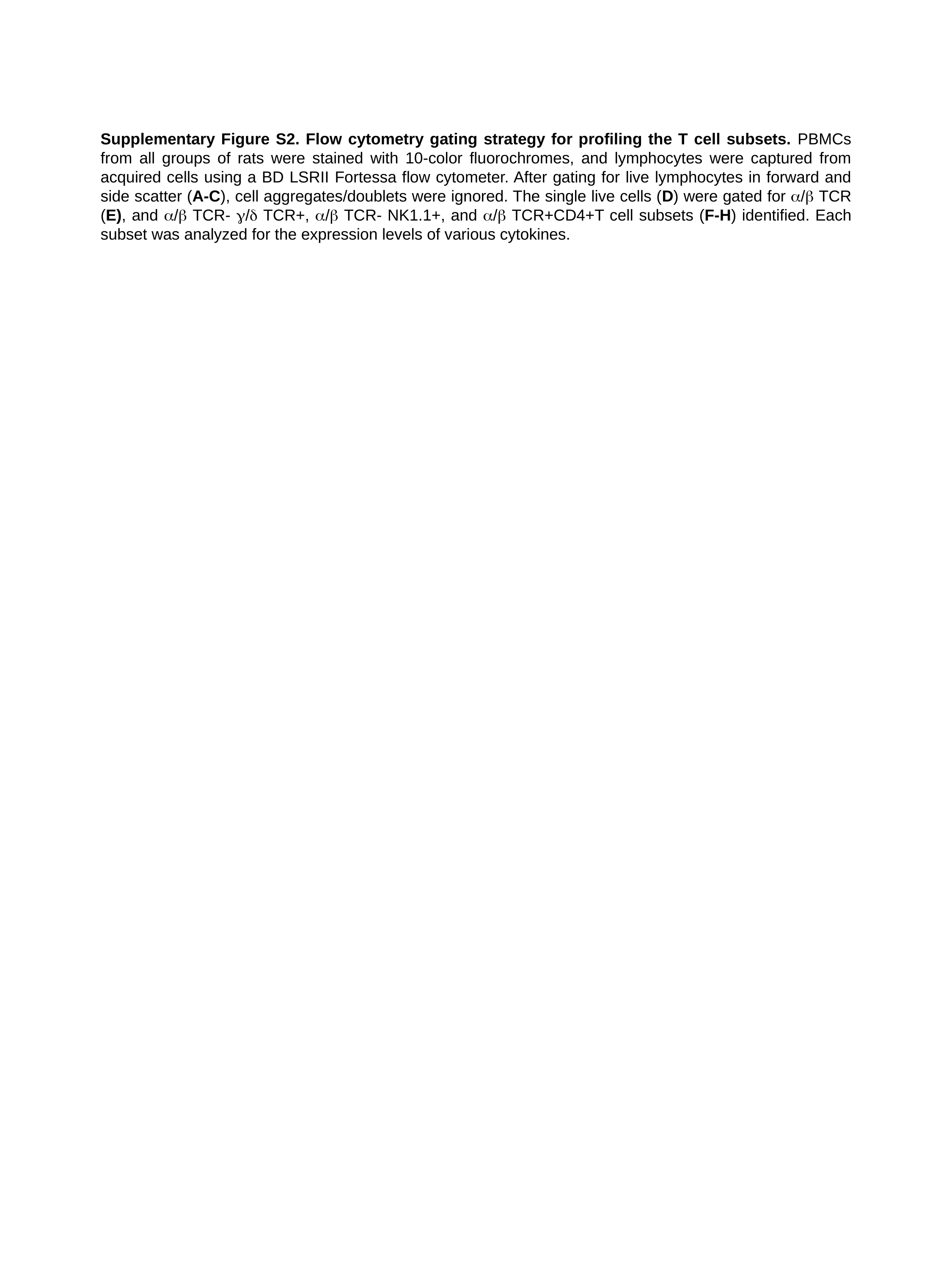

Supplementary Figure S2. Flow cytometry gating strategy for profiling the T cell subsets. PBMCs from all groups of rats were stained with 10-color fluorochromes, and lymphocytes were captured from acquired cells using a BD LSRII Fortessa flow cytometer. After gating for live lymphocytes in forward and side scatter (A-C), cell aggregates/doublets were ignored. The single live cells (D) were gated for α/β TCR (E), and α/β TCR- γ/ TCR+, α/β TCR- NK1.1+, and α/β TCR+CD4+T cell subsets (F-H) identified. Each subset was analyzed for the expression levels of various cytokines.

## Slide 3
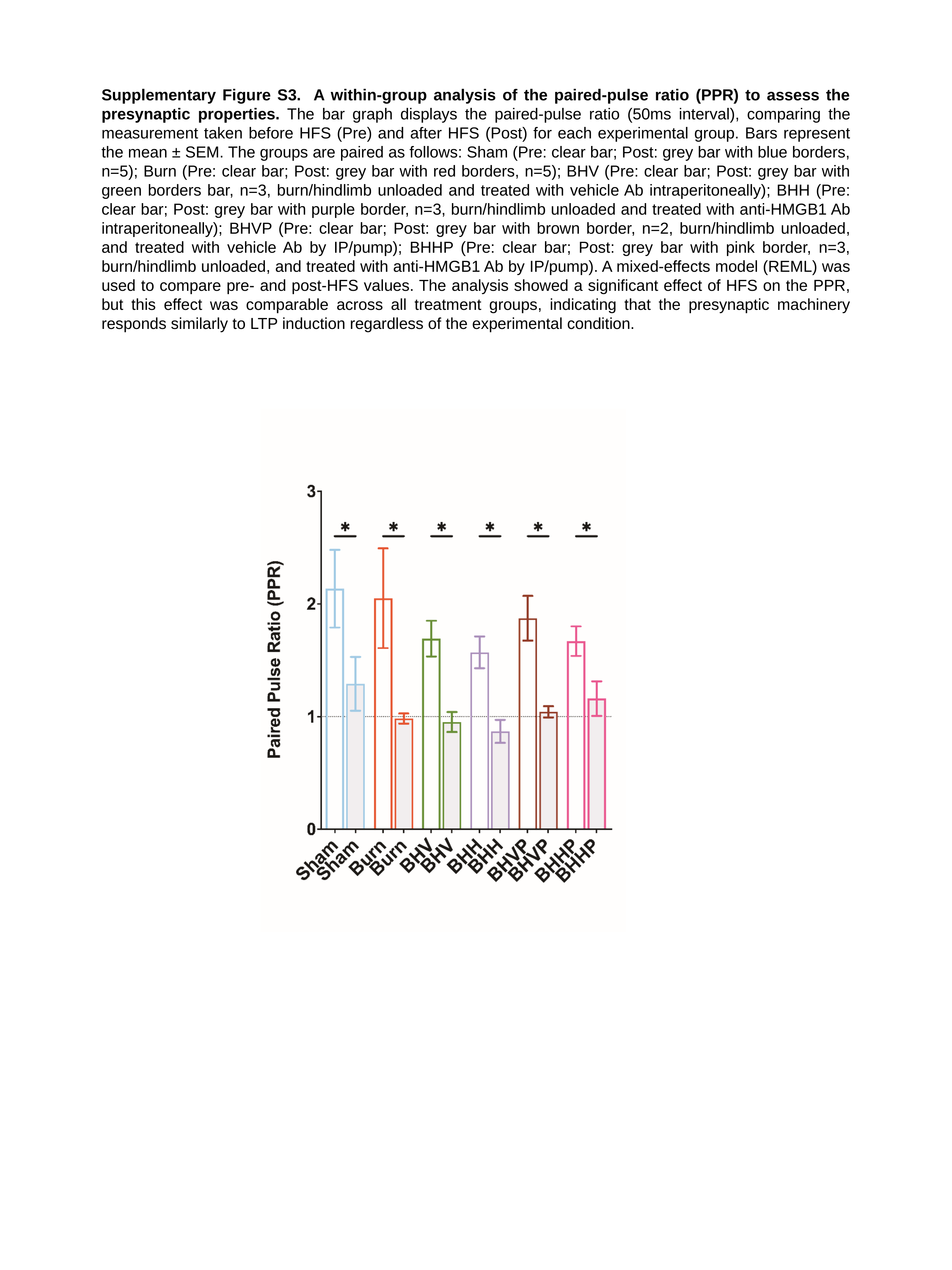

Supplementary Figure S3. A within-group analysis of the paired-pulse ratio (PPR) to assess the presynaptic properties. The bar graph displays the paired-pulse ratio (50ms interval), comparing the measurement taken before HFS (Pre) and after HFS (Post) for each experimental group. Bars represent the mean ± SEM. The groups are paired as follows: Sham (Pre: clear bar; Post: grey bar with blue borders, n=5); Burn (Pre: clear bar; Post: grey bar with red borders, n=5); BHV (Pre: clear bar; Post: grey bar with green borders bar, n=3, burn/hindlimb unloaded and treated with vehicle Ab intraperitoneally); BHH (Pre: clear bar; Post: grey bar with purple border, n=3, burn/hindlimb unloaded and treated with anti-HMGB1 Ab intraperitoneally); BHVP (Pre: clear bar; Post: grey bar with brown border, n=2, burn/hindlimb unloaded, and treated with vehicle Ab by IP/pump); BHHP (Pre: clear bar; Post: grey bar with pink border, n=3, burn/hindlimb unloaded, and treated with anti-HMGB1 Ab by IP/pump). A mixed-effects model (REML) was used to compare pre- and post-HFS values. The analysis showed a significant effect of HFS on the PPR, but this effect was comparable across all treatment groups, indicating that the presynaptic machinery responds similarly to LTP induction regardless of the experimental condition.

## Slide 4
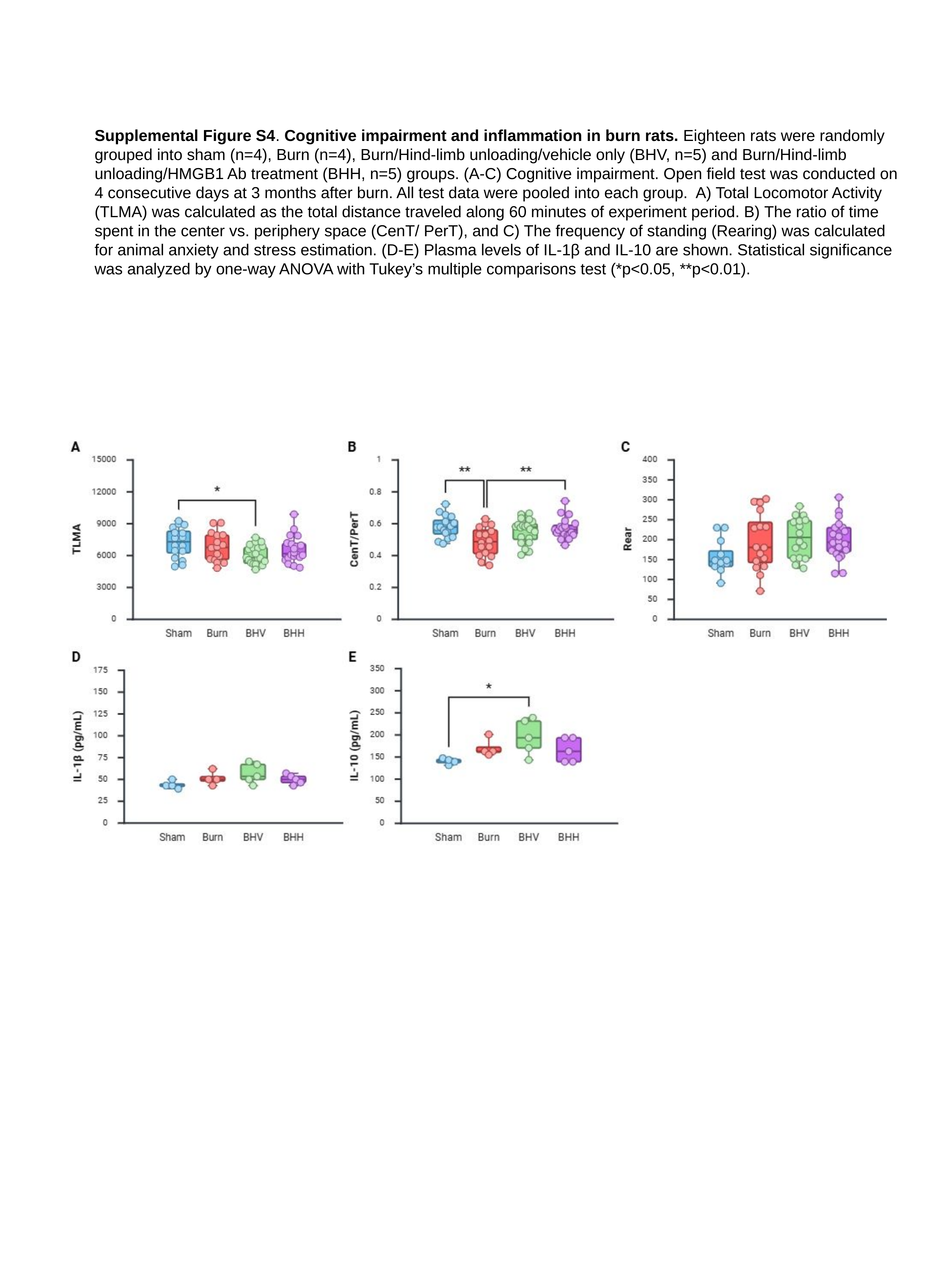

Supplemental Figure S4. Cognitive impairment and inflammation in burn rats. Eighteen rats were randomly grouped into sham (n=4), Burn (n=4), Burn/Hind-limb unloading/vehicle only (BHV, n=5) and Burn/Hind-limb unloading/HMGB1 Ab treatment (BHH, n=5) groups. (A-C) Cognitive impairment. Open field test was conducted on 4 consecutive days at 3 months after burn. All test data were pooled into each group. A) Total Locomotor Activity (TLMA) was calculated as the total distance traveled along 60 minutes of experiment period. B) The ratio of time spent in the center vs. periphery space (CenT/ PerT), and C) The frequency of standing (Rearing) was calculated for animal anxiety and stress estimation. (D-E) Plasma levels of IL-1β and IL-10 are shown. Statistical significance was analyzed by one-way ANOVA with Tukey’s multiple comparisons test (*p<0.05, **p<0.01).

## Slide 5
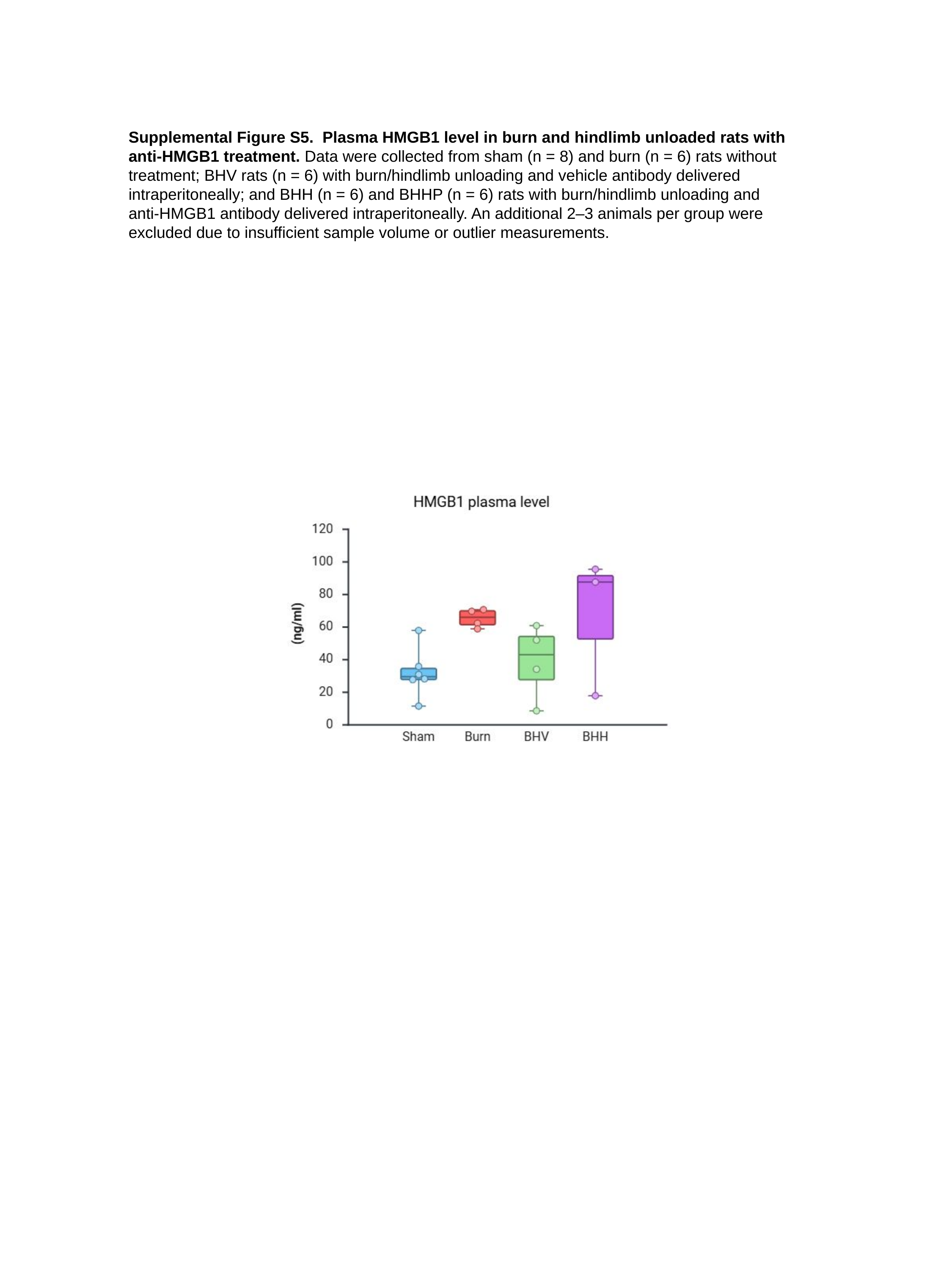

Supplemental Figure S5. Plasma HMGB1 level in burn and hindlimb unloaded rats with anti-HMGB1 treatment. Data were collected from sham (n = 8) and burn (n = 6) rats without treatment; BHV rats (n = 6) with burn/hindlimb unloading and vehicle antibody delivered intraperitoneally; and BHH (n = 6) and BHHP (n = 6) rats with burn/hindlimb unloading and anti‑HMGB1 antibody delivered intraperitoneally. An additional 2–3 animals per group were excluded due to insufficient sample volume or outlier measurements.
